# Supplementary material for: A cancer drug atlas enables synergistic targeting of independent drug vulnerabilities
Source: Nat Commun. 2020 Jun 10;11:2935. doi: 10.1038/s41467-020-16735-2 (PMC7287046; doi:10.1038/s41467-020-16735-2)
Supplement: Supplementary file 1 — Supplementary Information [file 41467_2020_16735_MOESM1_ESM.pdf]

Contents

Supplementary Figure 1 2
Supplementary Figure 1 (continued) 3
Supplementary Figure 2 4
Supplementary Figure 3 5
Supplementary Figure 4 6
Supplementary Figure 5 7
Legends to Supplementary figures 8
Supplementary references: curated synergy data 13

Supplementary Figure 1

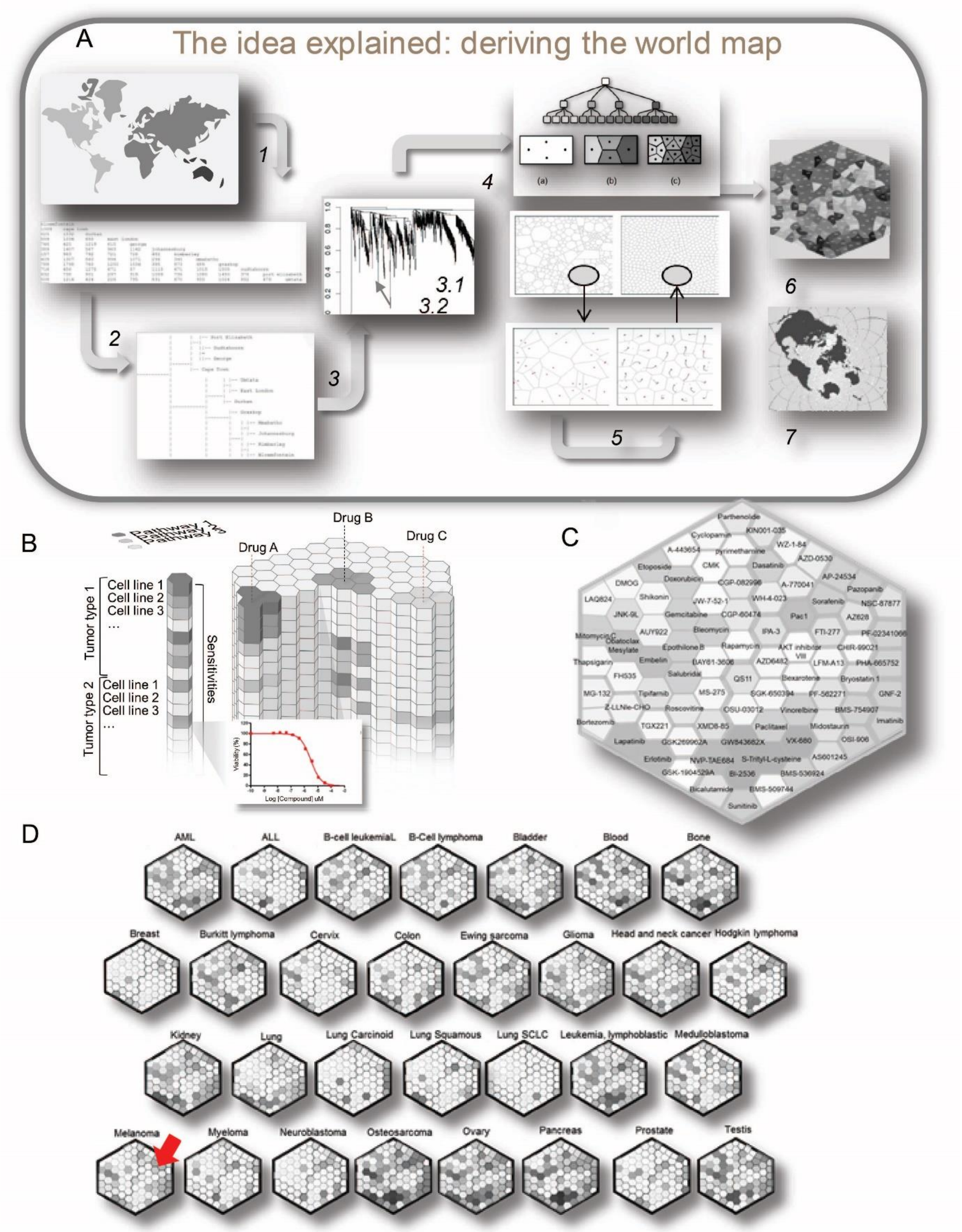

Supplementary Figure 1 (continued)

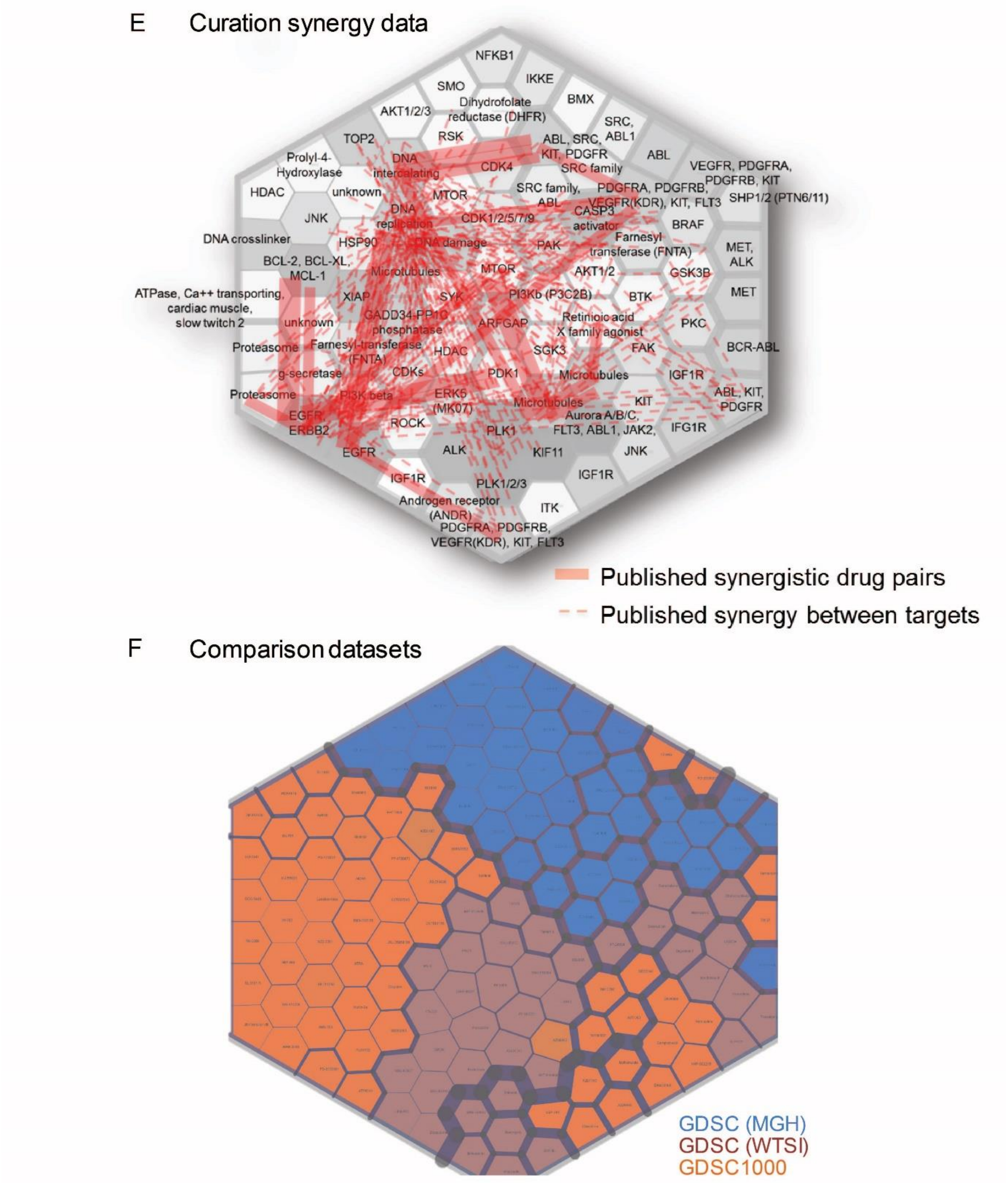

Supplementary Figure 2

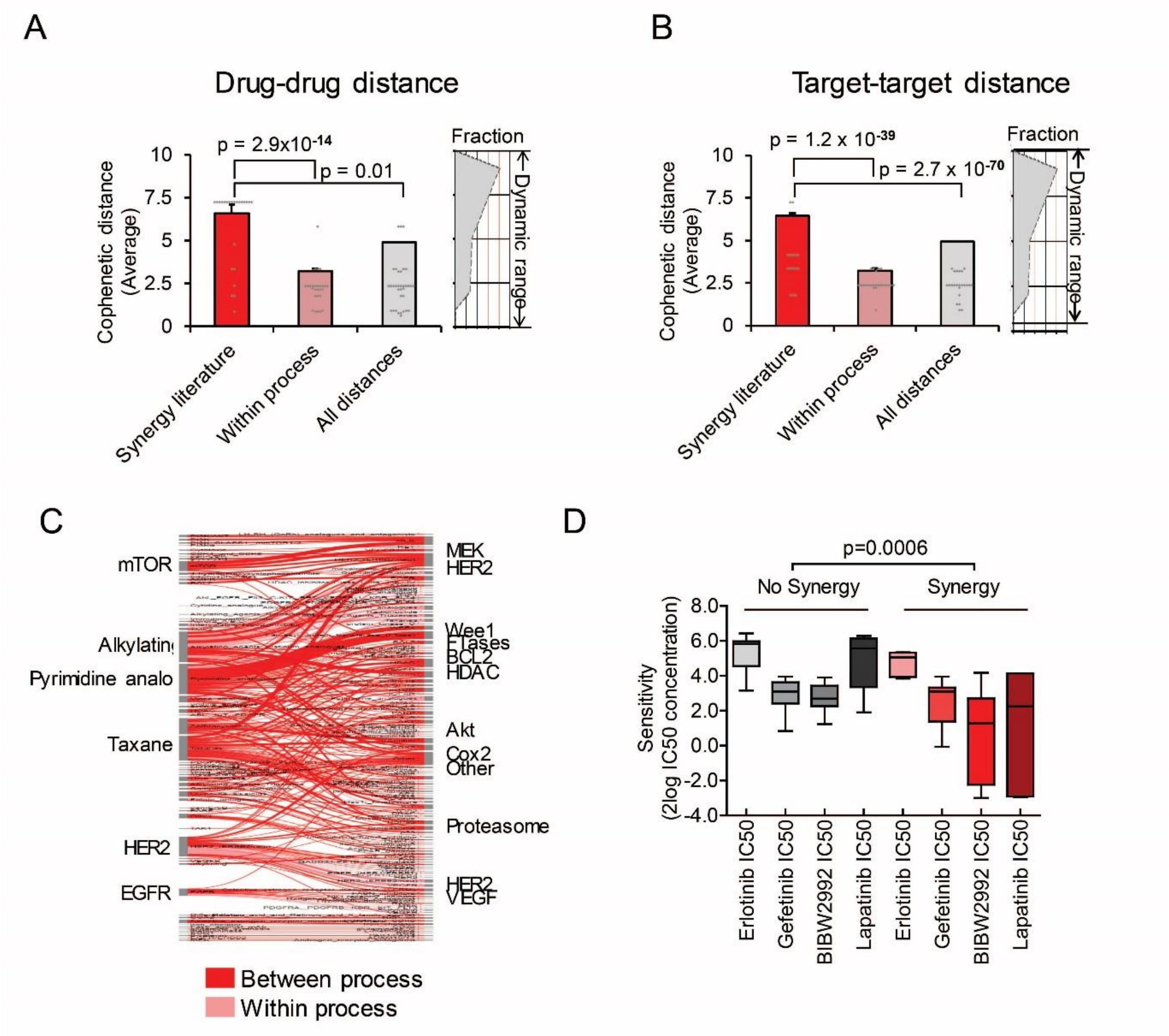

Supplementary Figure 3

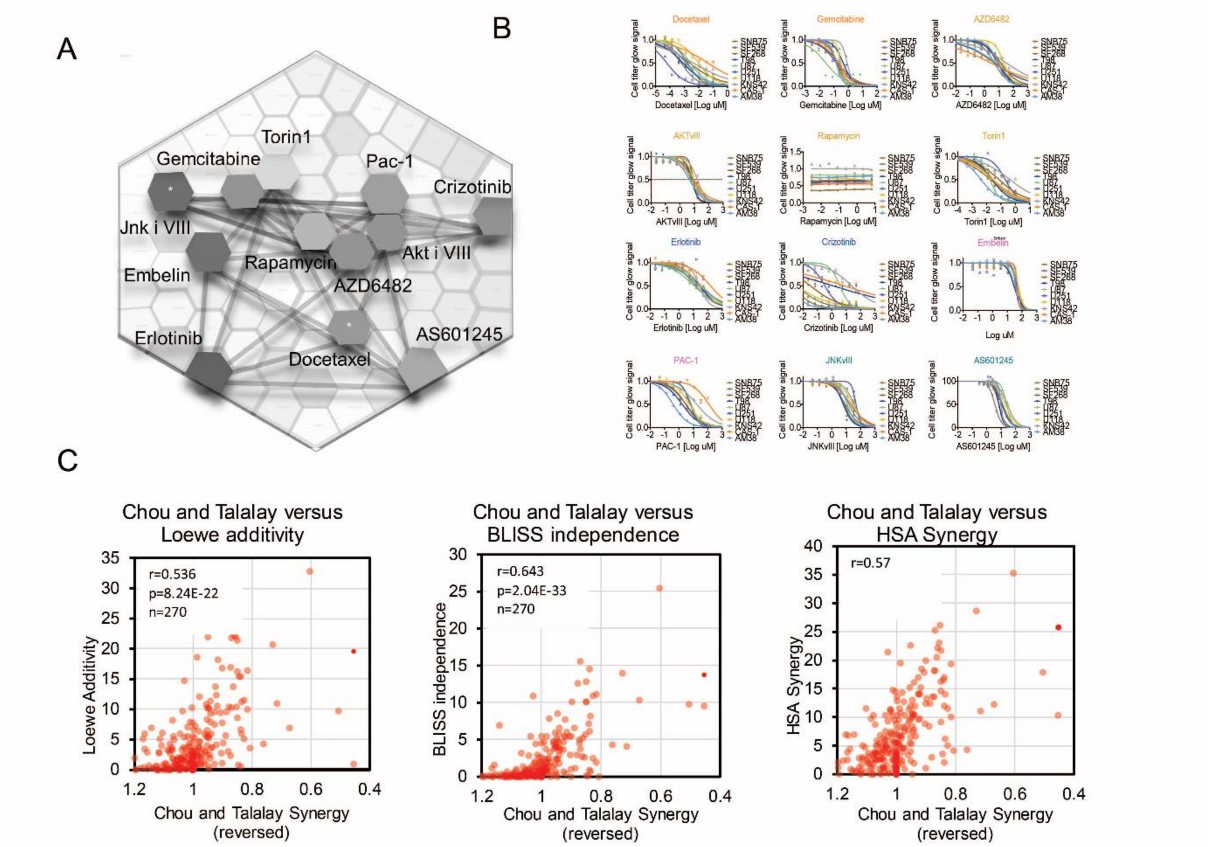

Correlation (Pearson)

	Chou^{rev}	LOEWE	BLISS	HSA
Chou^{rev}	NA	0.536	0.643	0.579
LOEWE	0.536	NA	0.644	0.901
BLISS	0.643	0.644	NA	0.691
HSA	0.579	0.901	0.691	NA

p-value

	Chou^{rev}	LOEWE	BLISS	HSA
Chou^{rev}	NA	8.24E-22	3.04E-33	7.14E-26
LOEWE	8.24E-22	NA	2.56E-33	7.8E-100
BLISS	3.04E-33	2.56E-33	NA	4.62E-40
HSA	7.14E-26	7.8E-100	4.62E-40	NA

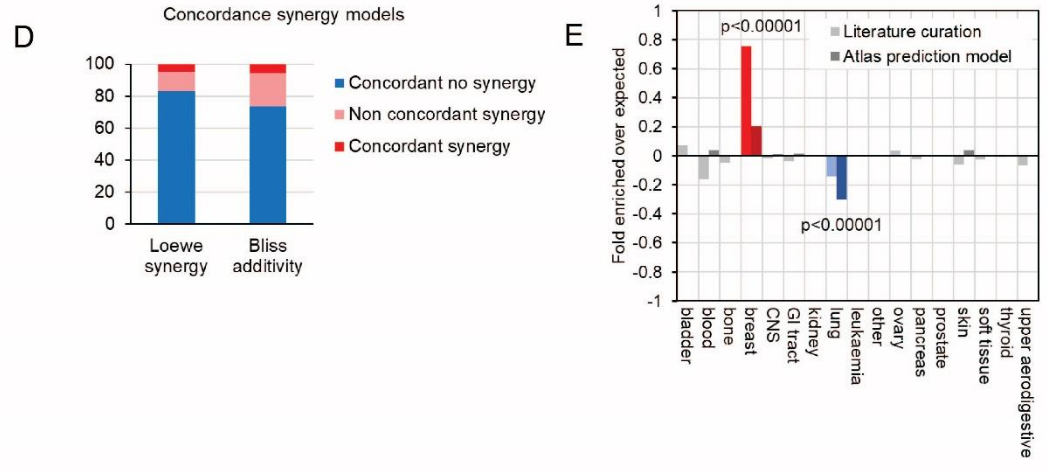

Supplementary Figure 4

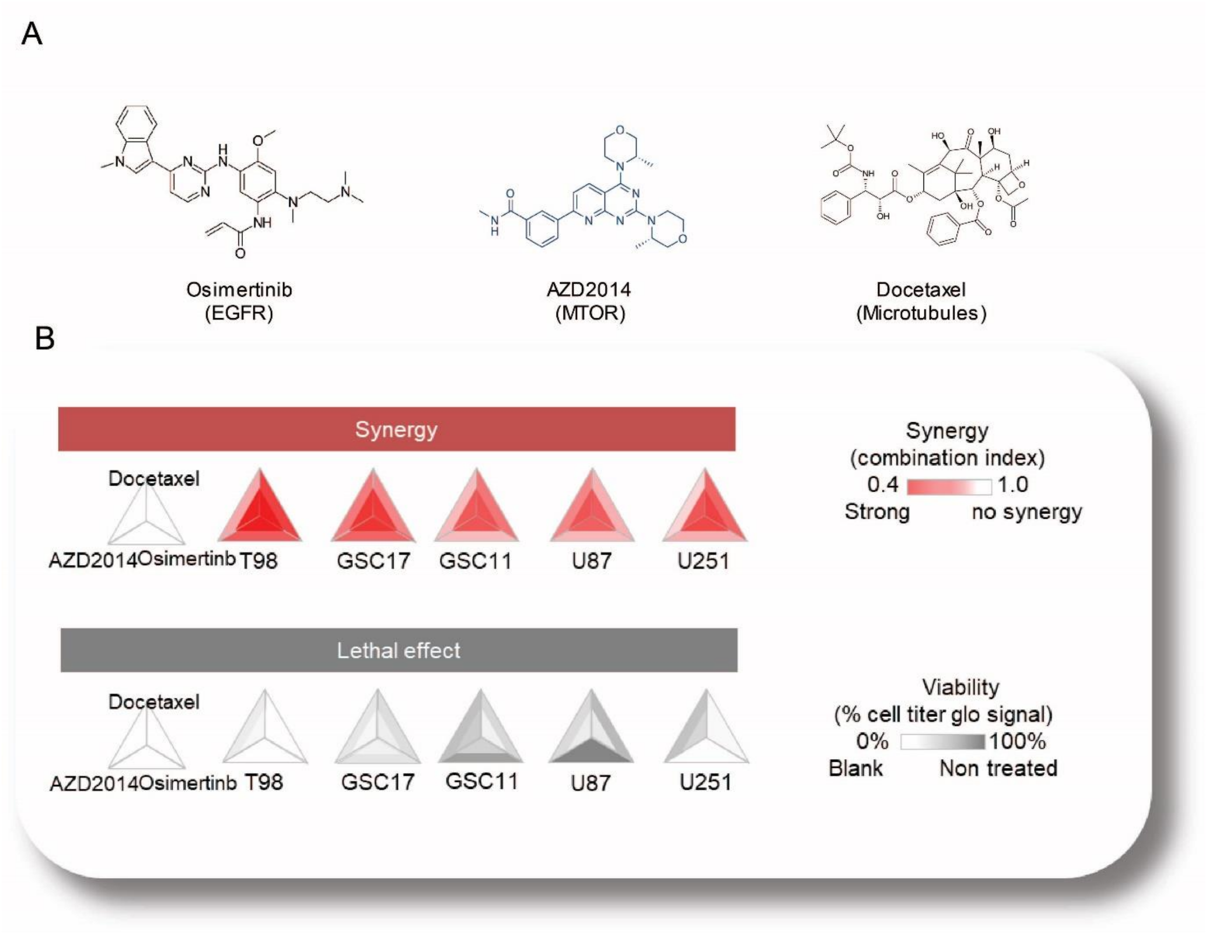

Supplementary Figure 5

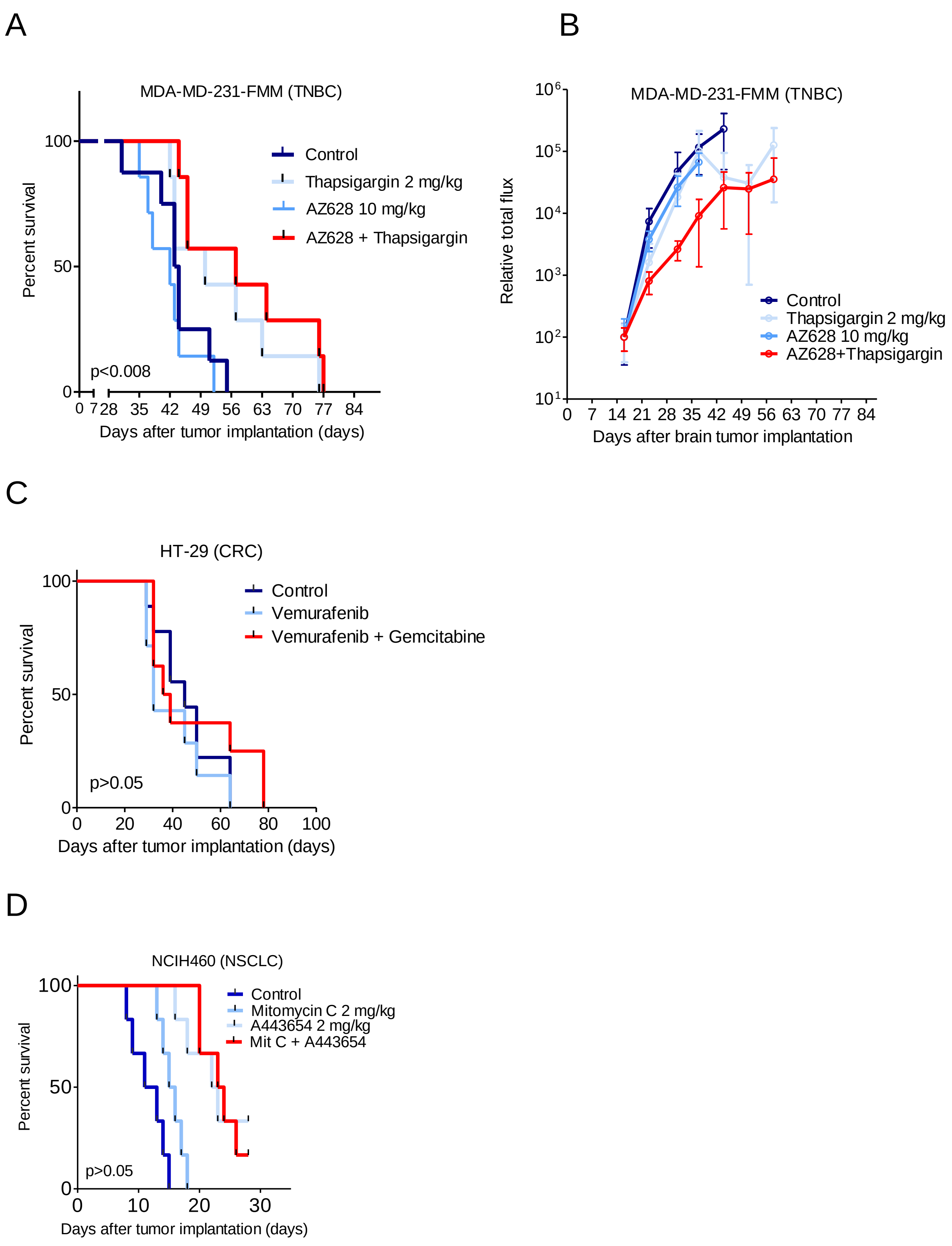

Legends to Supplementary figures

Supplementary Figure 1. Deriving a map based onto topological data (A) Schematic visualization of the method used to create the drug-atlas. Here an example of the topography of the world-map is shown. Steps in the algorithm are (1) For all n elements∈ dataspace D calculate the n x n similarity matrix M. (2) Hierarchically cluster M → binary cluster tree T, (3) Calculate branch grouping threshold based on percentage clusters formed at tree depth d (4) Recursively layout branches: apply weighted Fast Voronoi Layout (FVL) per branch, (5) Smoothen Va map by re-applying FVL while maintaining relative positions. Comparison of end result (6) with the world map in a conformal projection (7). Dark greys reflect high level splits in the cluster tree and relate to the continents on the world map. (B) Schematic visualization of the drug atlas. In the example shown, drug A and B show no overlap in sensitivity, in contrast to drug C which shows no overlap with drug A. After clustering and projection onto the map, drug A and drug B are in relative close vicinity and drug C is most distally related to drug A. (C) The resulting drug atlas can be considered as a generalized map of cancer drug responses since it is based on more than 600 cell lines representing an array of tumor types and genetic variations. (D) The drug-atlas can be used to annotate the relative drug sensitivity where the average drug sensitivity per tumor-type is visualized by the intensity of the drug. The dynamic range was set based on the overall dynamic range for each drug. Red arrow: high BRAF/MEK sensitivity in melanoma patients due to frequent BRAFV600 mutations. For comparison of different dose response encyclopedias, see also **Quality control** section under the Supplementary Methods. (E) Detailed view of the curated synergy data depicted onto the drug atlas

showing targets. (F) Distal clustering of drug dose-response data of the CCLE and GDSC encyclopedia shows limited experimental comparability of the experiments. The GDSC (MGH) data shows the most consistent clustering and was chosen to create the drug atlas.

Supplementary Figure 2. Target combinations and distance relation of curated data

(A) Histogram showing the average cophenetic distance of synergistic drug pairs (red) compared to within-pathway distances (pink) and all possible distances (grey), showing that synergistic drugs show a significant higher distance compared to the average of all possible distances. To calculate the cluster distance, average clustering was used here. The dynamic window of all possible drug-distances is shown on the right of the histogram. (B) Histogram showing the average cophenetic distance of synergistic drug pairs (red) compared to within pathway distance (pink) based on the targets of the respective drugs, again showing that synergistic drug pairs have a significantly higher distance. (C) Sankey diagram showing the molecular targets of the curated synergistic drugs. The most common targets are highlighted. (D) In case synergy was found in breast cancer, significantly more sensitivity was seen for EGFR/HER2 inhibitors. p-values A, B, D student t-test (one-sided). Curated drug-drug distances synergistic drug-pairs n=81, all drug-drug distances: n=8515; Within pathway distances, n=235; target-target synergistic pairs n=193. Sensivity data of no synergy versus synergy: box-and-whiskers plot, minimum, 25th percentile, median, 75th percentile, and maximum; Erlotinib n=7 versus 5; Gefitinib n=13 versus 12; BIBW2992 n=16 versus 13; Lapatinib n=7 versus 5.

Supplementary Figure 3. Large cophenetic distance and high sensitivity can predict synergy (A) 30 drug pairs were selected based on the high sensitivity in GBM as well as a relative high cophenetic distance as shown from their corresponding position on the drug-atlas. (B) Dose-response data of 9 cell lines when the 12 selected drugs were applied. These data were used to determine maximal concentrations (IC50s) that were used for drug combination experiments. (C) Scatterplot showing an correlation between the Chou and Talalay Combination Index versus other drug combination effect metrics (Loewe synergy, Bliss additivity and HSA). Also all cross correlations are shown in the correlation table with the according p-value table. (D) Histogram showing the concordance between different synergy models. Either high levels or absence of synergy show a good concordance and weaker interactions provide a non-concordant outcome of the score. Thresholds for synergy were estimated based on interpolating the correlation plot between the different type of synergies, CI: 0.8; Loewe 7.8 and Bliss 2.5. (E) Histogram showing that drug synergies are found more frequently than expected in breast cancer, both for curated literature data (light red) as well as drug atlas mediated prediction of drug-combinations (dark red). In contrast, synergies in lung tumors are less frequently found than expected. Drug dose-response experiments were performed in triplicate and repeated independently at least once. For synergy metrics: p-value is the Pearson correlation significance of n=270 drug combinations. p-value in (E) based on Chi2 test, n between 1 and 218 synergy observations per tumour type (literature) and between 1168 to 5990 predicted synergies per tumor type, missing data were taken into account.

Supplementary Figure 4. Validation of synergistic therapy of three drugs that penetrate the brain (A) Structures of drugs chosen for the study. (B) *In vitro* efficacy of the three compounds when tested onto 5 cell lines/primary cultures that previously showed synergy. Synergy is shown in the upper panel and the according viability as measured after three days of drug treatment is shown on the lower panel.

Supplementary Figure 5. In vivo validation of predicted combination therapies. (A) Kaplan Meier survival analysis of tumor growth after orthotopic transplantation of Fluc tagged MDA-MD-231 triple negative breast cancer (TNBC) cells. Tumors were engrafted for one week and then treated with Thapsigargin, AZ628 or their combination in relation to an control group that was treated with carrier only, showing a significant survival benefit of the combination. (B) Measurement of averages of luciferase-activity in the orthotopically transplanted tumors showed a strong efficacy of the drug combination, resulting in a synergistic response as indicated by the combination-index between 0.61 at day 23 to 0.15 at day 37. (C) Kaplan Meier survival analysis of tumor growth after orthotopic transplantation of Fluc tagged HT-29 colorectal cancer (CRC) cells. Tumors were engrafted for one week then treated with Vemurafenib, Gemcitabine or their combination in relation to an control treated with carrier only, resulting in small but non-significant survival benefit for the combination. (D) Kaplan Meier survival analysis of tumor growth after orthotopic transplantation of Fluc tagged NCI-H460 non-small cell lung cancer (NSCLC) cells. Tumors were engrafted for one week then treated with Mitomycin C, A443654 or their combination in relation to an control treated with carrier only, resulting in a small but non-significant survival benefit. For all experiments, luciferase levels were normalized to

the monotherapy levels of one week after injection. Toxicity monitoring consisted of assessment of body weight, hematopoietic-, liver- and brain-toxicity. p-value: t-test (one-sided) of the median survival. Number of mice per group: MDA-MD-231, n=7; HT-29, n=5; NCI-H460, n=6.

Supplementary references: curated synergy data

1. Aarts, M., Sharpe, R., Garcia-Murillas, I., Gevensleben, H., Hurd, M. S., Shumway, S. D., … Turner, N. C. (2012). Forced mitotic entry of S-phase cells as a therapeutic strategy induced by inhibition of WEE1. *Cancer Discovery*, *2*(6), 524–539. http://doi.org/10.1158/2159-8290.CD-11-0320
2. Adjei, A. A., Budihardjo, I. I., Rowinsky, E. K., Kottke, T. J., Svingen, P. A., Buckwalter, C. A., … Kaufmann, S. H. (1997). Cytotoxic synergy between pyrazoloacridine (NSC 366140) and cisplatin in vitro: inhibition of platinum-DNA adduct removal. *Clinical Cancer Research : An Official Journal of the American Association for Cancer Research*, *3*(5), 761–770.
3. Adjei, A. A., Davis, J. N., Bruzek, L. M., Erlichman, C., & Kaufmann, S. H. (2001). Synergy of the protein farnesyltransferase inhibitor SCH66336 and cisplatin in human cancer cell lines. *Clinical Cancer Research : An Official Journal of the American Association for Cancer Research*, *7*(5), 1438–1445.
4. Aghi, M., Rabkin, S., & Martuza, R. L. (2006). Effect of chemotherapy-induced DNA repair on oncolytic herpes simplex viral replication. *Journal of the National Cancer Institute*, *98*(1), 38–50. http://doi.org/10.1093/jnci/djj003
5. Ahmed, S. M. U., Wu, X., Jin, X., Zhang, X., Togo, Y., Suzuki, T., … Kakehi, Y. (2015). Synergistic induction of apoptosis by mapatumumab and anthracyclines in human bladder cancer cells. *Oncology Reports*, *33*(2), 566–72. http://doi.org/10.3892/or.2014.3654
6. Allen, W. L., McLean, E. G., Boyer, J., McCulla, A., Wilson, P. M., Coyle, V., … Johnston, P. G. (2007). The role of spermidine/spermine N1-acetyltransferase in determining response to chemotherapeutic agents in colorectal cancer cells. *Molecular Cancer Therapeutics*, *6*(1), 128–137. http://doi.org/10.1158/1535-7163.MCT-06-0303

7. An, J., Chervin, A. S., Nie, A., Ducoff, H. S., & Huang, Z. (2007). Overcoming the radioresistance of prostate cancer cells with a novel Bcl-2 inhibitor. *Oncogene*, *26*(5), 652–661. http://doi.org/10.1038/sj.onc.1209830

8. Anderson, T. R., & Slotkin, T. A. (1975). Maturation of the adrenal medulla--IV. Effects of morphine. *Biochemical Pharmacology*, *24*(16), 1469–1474.

9. Arantes-Rodrigues, R., Pinto-Leite, R., Fidalgo-Goncalves, L., Palmeira, C., Santos, L., Colaco, A., & Oliveira, P. (2013). Synergistic effect between cisplatin and sunitinib malate on human urinary bladder-cancer cell lines. *BioMed Research International*, *2013*, 791406. http://doi.org/10.1155/2013/791406

10. Argiris, A., Wang, C.-X., Whalen, S. G., & DiGiovanna, M. P. (2004). Synergistic interactions between tamoxifen and trastuzumab (Herceptin). *Clinical Cancer Research : An Official Journal of the American Association for Cancer Research*, *10*(4), 1409–1420.

11. Arias-Romero, L. E., Villamar-Cruz, O., Huang, M., Hoeflich, K. P., & Chernoff, J. (2013). Pak1 kinase links ErbB2 to beta-catenin in transformation of breast epithelial cells. *Cancer Research*, *73*(12), 3671–3682. http://doi.org/10.1158/0008-5472.CAN-12-4453

12. Azrak, R. G., Frank, C. L., Ghadersohi, A., & Rustum, Y. M. (2008). Silencing survivin results in synergy between methylseleninic acid and paclitaxel against skov3 ovarian cancer cells. *Cancer Biology & Therapy*, *7*(12), 1901–1908.

13. Azrak, R. G., Yu, J., Pendyala, L., Smith, P. F., Cao, S., Li, X., … Rustum, Y. M. (2005). Irinotecan pharmacokinetic and pharmacogenomic alterations induced by methylselenocysteine in human head and neck xenograft tumors. *Molecular Cancer Therapeutics*, *4*(5), 843–854. http://doi.org/10.1158/1535-7163.MCT-04-0315

14. Bali, P., Pranpat, M., Swaby, R., Fiskus, W., Yamaguchi, H., Balasis, M., … Bhalla, K. (2005). Activity of suberoylanilide hydroxamic Acid against human breast cancer cells with amplification of her-2. *Clinical Cancer Research : An Official Journal of the American Association for Cancer Research*, *11*(17), 6382–6389. http://doi.org/10.1158/1078-0432.CCR-05-0344

15. Balzarotti, M., Ciusani, E., Calatozzolo, C., Croci, D., Boiardi, A., & Salmaggi, A. (2004). Effect of association of temozolomide with other chemotherapic agents on cell growth inhibition in glioma cell lines. *Oncology Research*, *14*(7–8), 325–330.

16. Bartholomeusz, C., Yamasaki, F., Saso, H., Kurisu, K., Hortobagyi, G. N., & Ueno, N. T. (2011). Gemcitabine Overcomes Erlotinib Resistance in EGFR-Overexpressing Cancer Cells through Downregulation of Akt. *Journal of Cancer*, *2*, 435–442.

17. Bauer, J. A., Ye, F., Marshall, C. B., Lehmann, B. D., Pendleton, C. S., Shyr, Y., … Pietenpol, J. A. (2010). RNA interference (RNAi) screening approach identifies agents that enhance paclitaxel activity in breast cancer cells. *Breast Cancer Research : BCR*, *12*(3), R41. http://doi.org/10.1186/bcr2595

18. Becker MN, Wu KJ, Marlow LA, Kreinest PA, Vonroemeling CA, Copland JA, Williams CR, The combination of an mTORc1/TORc2 inhibitor with lapatinib is synergistic in bladder cancer in vitro. Urol Oncol. 2014 Apr;32(3):317-26.

19. Biswas, R., Ahn, J.-C., & Kim, J.-S. (2015). Sulforaphene Synergistically Sensitizes Cisplatin via Enhanced Mitochondrial Dysfunction and PI3K/PTEN Modulation in Ovarian Cancer Cells. *Anticancer Research*, *35*(7), 3901–3908.

20. Bjorkman, M., Iljin, K., Halonen, P., Sara, H., Kaivanto, E., Nees, M., & Kallioniemi, O. P. (2008). Defining the molecular action of HDAC inhibitors and synergism with androgen

deprivation in ERG-positive prostate cancer. *International Journal of Cancer*, *123*(12), 2774–2781. http://doi.org/10.1002/ijc.23885

21. Bodo, J., Chovancova, J., Hunakova, L., & Sedlak, J. (2005). Enhanced sensitivity of human ovarian carcinoma cell lines A2780 and A2780/CP to the combination of cisplatin and synthetic isothiocyanate ethyl 4-isothiocyanatobutanoate. *Neoplasma*, *52*(6), 510–516.
22. Budman, D. R., Calabro, A., & Kreis, W. (2002). Synergistic and antagonistic combinations of drugs in human prostate cancer cell lines in vitro. *Anti-Cancer Drugs*, *13*(10), 1011–1016.
23. Budman, D. R., Calabro, A., Rosen, L., & Lesser, M. (2012). Identification of unique synergistic drug combinations associated with downexpression of survivin in a preclinical breast cancer model system. *Anti-Cancer Drugs*, *23*(3), 272–279.
24. Budman, D. R., Soong, R., Calabro, A., Tai, J., & Diasio, R. (2006). Identification of potentially useful combinations of epidermal growth factor receptor tyrosine kinase antagonists with conventional cytotoxic agents using median effect analysis. *Anti-Cancer Drugs*, *17*(8), 921–928. http://doi.org/10.1097/01.cad.0000224457.36522.60
25. Cardillo, T. M., Trisal, P., Arrojo, R., Goldenberg, D. M., & Chang, C.-H. (2013). Targeting both IGF-1R and mTOR synergistically inhibits growth of renal cell carcinoma in vitro. *BMC Cancer*, *13*, 170. http://doi.org/10.1186/1471-2407-13-170
26. Cardoso, F., Durbecq, V., Laes, J.-F., Badran, B., Lagneaux, L., Bex, F., … Sotiriou, C. (2006). Bortezomib (PS-341, Velcade) increases the efficacy of trastuzumab (Herceptin) in HER-2-positive breast cancer cells in a synergistic manner. *Molecular Cancer Therapeutics*, *5*(12), 3042–3051. http://doi.org/10.1158/1535-7163.MCT-06-0104

27. Chan, M., Gravel, M., Bramoulle, A., Bridon, G., Avizonis, D., Shore, G. C., & Roulston, A. (2014). Synergy between the NAMPT inhibitor GMX1777(8) and pemetrexed in non-small cell lung cancer cells is mediated by PARP activation and enhanced NAD consumption. *Cancer Research*, *74*(21), 5948–5954. http://doi.org/10.1158/0008-5472.CAN-14-0809

28. Chan, S.-E., Lai, H.-W., Su, C.-C., Kuo, S.-J., Chien, S.-Y., Lin, H.-Y., & Chen, D.-R. (2011). Effect of Supplementation of Tanshinone IIA and Sodium Tanshinone IIA Sulfonate on the Anticancer Effect of Epirubicin: An In Vitro Study. *Evidence-Based Complementary and Alternative Medicine : eCAM*, *2011*, 841564. http://doi.org/10.1155/2011/841564

29. Chan, T. C., & Howell, S. B. (1989). Unexpected synergy between N-phosphonacetyl-L-aspartate and cytidine against human tumor cells. *European Journal of Cancer & Clinical Oncology*, *25*(4), 721–727.

30. Chen, S.-M., Guo, C.-L., Shi, J.-J., Xu, Y.-C., Chen, Y., Shen, Y.-Y., … Meng, L.-H. (2014). HSP90 inhibitor AUY922 abrogates up-regulation of RTKs by mTOR inhibitor AZD8055 and potentiates its antiproliferative activity in human breast cancer. *International Journal of Cancer*, *135*(10), 2462–2474. http://doi.org/10.1002/ijc.28880

31. Chen, S.-Z., Jiang, M., & Zhen, Y. (2005). HERG K+ channel expression-related chemosensitivity in cancer cells and its modulation by erythromycin. *Cancer Chemotherapy and Pharmacology*, *56*(2), 212–220. http://doi.org/10.1007/s00280-004-0960-5

32. Chitambar, C. R., Matthaeus, W. G., Antholine, W. E., Graff, K., & O'Brien, W. J. (1988). Inhibition of leukemic HL60 cell growth by transferrin-gallium: effects on ribonucleotide reductase and demonstration of drug synergy with hydroxyurea. *Blood*, *72*(6), 1930–1936.

33. Ciesielski, M. J., & Fenstermaker, R. A. (1999). Synergistic cytotoxicity, apoptosis and protein-linked DNA breakage by etoposide and camptothecin in human U87 glioma cells: dependence on tyrosine phosphorylation. *Journal of Neuro-Oncology*, *41*(3), 223–234.

34. da Silva, G. N., de Castro Marcondes, J. P., de Camargo, E. A., da Silva Passos Júnior, G. A., Sakamoto-Hojo, E. T., & Salvadori, D. M. F. (2010). Cell cycle arrest and apoptosis in TP53 subtypes of bladder carcinoma cell lines treated with cisplatin and gemcitabine. *Experimental Biology and Medicine (Maywood, N.J.)*, *235*(7), 814–24. http://doi.org/10.1258/ebm.2010.009322

35. Deng, R., Tang, J., Xia, L.-P., Li, D.-D., Zhou, W.-J., Wang, L.-L., … Zhu, X.-F. (2009). ExcisaninA, a diterpenoid compound purified from Isodon MacrocalyxinD, induces tumor cells apoptosis and suppresses tumor growth through inhibition of PKB/AKT kinase activity and blockade of its signal pathway. *Molecular Cancer Therapeutics*, *8*(4), 873–882. http://doi.org/10.1158/1535-7163.MCT-08-1080

36. Donnelly, S. M., Paplomata, E., Peake, B. M., Sanabria, E., Chen, Z., & Nahta, R. (2014). P38 MAPK contributes to resistance and invasiveness of HER2- overexpressing breast cancer. *Current Medicinal Chemistry*, *21*(4), 501–510.

37. Duangjai, A., Luo, K., Zhou, Y., Yang, J., & Kopecek, J. (2014). Combination cytotoxicity of backbone degradable HPMA copolymer gemcitabine and platinum conjugates toward human ovarian carcinoma cells. *European Journal of Pharmaceutics and Biopharmaceutics : Official Journal of Arbeitsgemeinschaft Fur Pharmazeutische Verfahrenstechnik e.V*, *87*(1), 187–196. http://doi.org/10.1016/j.ejpb.2013.11.008

38. Edeline, J., Coulouarn, C., Crouzet, L., Pracht, M., Lepareur, N., Clement, B., & Garin, E. (2015). Gemcitabine and Oxaliplatin, but Not Sorafenib or Paclitaxel, Have a

Synergistic Effect with Yttrium-90 in Reducing Hepatocellular Carcinoma and Cholangiocarcinoma Cell Line Viability. *Journal of Vascular and Interventional Radiology : JVIR*, *26*(12), 1874–78.e2. http://doi.org/10.1016/j.jvir.2015.06.032

39. Edelman, M. J., Quam, H., & Mullins, B. (2001). Interactions of gemcitabine, carboplatin and paclitaxel in molecularly defined non-small-cell lung cancer cell lines. *Cancer Chemotherapy and Pharmacology*, *48*(2), 141–144.

40. Elkady, A. I., Hussein, R. A. E. H., & Abu-Zinadah, O. A. (2014). Effects of crude extracts from medicinal herbs Rhazya stricta and Zingiber officinale on growth and proliferation of human brain cancer cell line in vitro. *BioMed Research International*, *2014*, 260210. http://doi.org/10.1155/2014/260210

41. Esparis-Ogando, A., Ocana, A., Rodriguez-Barrueco, R., Ferreira, L., Borges, J., & Pandiella, A. (2008). Synergic antitumoral effect of an IGF-IR inhibitor and trastuzumab on HER2-overexpressing breast cancer cells. *Annals of Oncology : Official Journal of the European Society for Medical Oncology / ESMO*, *19*(11), 1860–1869. http://doi.org/10.1093/annonc/mdn406

42. Fang, X., Zheng, C., Liu, Z., Ekman, P., & Xu, D. (2004). Enhanced sensitivity of prostate cancer DU145 cells to cisplatinum by 5-aza-2’-deoxycytidine. *Oncology Reports*, *12*(3), 523–526.

43. Farivar-Mohseni, H., Kandzari, S. J., Zaslau, S., Riggs, D. R., Jackson, B. J., & McFadden, D. W. (2004). Synergistic effects of Cox-1 and -2 inhibition on bladder and prostate cancer in vitro. *American Journal of Surgery*, *188*(5), 505–510. http://doi.org/10.1016/j.amjsurg.2004.07.025

44. Fehlauer, F., Muench, M., Rades, D., Stalpers, L. J. A., Leenstra, S., van der Valk, P., … Sminia, P. (2005). Effects of irradiation and cisplatin on human glioma spheroids: inhibition of cell proliferation and cell migration. *Journal of Cancer Research and Clinical Oncology*, *131*(11), 723–732. http://doi.org/10.1007/s00432-005-0014-3

45. Feleszko, W., Jalili, A., Olszewska, D., Mlynarczuk, I., Grzela, T., Giermasz, A., & Jakobisiak, M. (2002). Synergistic interaction between highly specific cyclooxygenase-2 inhibitor, MF-tricyclic and lovastatin in murine colorectal cancer cell lines. *Oncology Reports*, *9*(4), 879–885.

46. Finn, R. S., Dering, J., Conklin, D., Kalous, O., Cohen, D. J., Desai, A. J., … Slamon, D. J. (2009). PD 0332991, a selective cyclin D kinase 4/6 inhibitor, preferentially inhibits proliferation of luminal estrogen receptor-positive human breast cancer cell lines in vitro. *Breast Cancer Research : BCR*, *11*(5), R77. http://doi.org/10.1186/bcr2419

47. Flowers, M., Fabrias, G., Delgado, A., Casas, J., Abad, J. L., & Cabot, M. C. (2012). C6-ceramide and targeted inhibition of acid ceramidase induce synergistic decreases in breast cancer cell growth. *Breast Cancer Research and Treatment*, *133*(2), 447–458. http://doi.org/10.1007/s10549-011-1768-8

48. Garcia-Recio, S., Fuster, G., Fernandez-Nogueira, P., Pastor-Arroyo, E. M., Park, S. Y., Mayordomo, C., … Almendro, V. (2013). Substance P autocrine signaling contributes to persistent HER2 activation that drives malignant progression and drug resistance in breast cancer. *Cancer Research*, *73*(21), 6424–6434. http://doi.org/10.1158/0008-5472.CAN-12-4573

49. Gaspar, N., Marshall, L., Perryman, L., Bax, D. A., Little, S. E., Viana-Pereira, M., … Jones, C. (2010). MGMT-independent temozolomide resistance in pediatric glioblastoma cells

associated with a PI3-kinase-mediated HOX/stem cell gene signature. *Cancer Research*, *70*(22), 9243–9252. http://doi.org/10.1158/0008-5472.CAN-10-1250

50. Gayle, S. S., Arnold, S. L. M., O'Regan, R. M., & Nahta, R. (2012). Pharmacologic inhibition of mTOR improves lapatinib sensitivity in HER2-overexpressing breast cancer cells with primary trastuzumab resistance. *Anti-Cancer Agents in Medicinal Chemistry*, *12*(2), 151–162.

51. Gharehbaghi, K., Szekeres, T., Yalowitz, J. A., Fritzer-Szekeres, M., Pommier, Y. G., & Jayaram, H. N. (2000). Sensitizing human colon carcinoma HT-29 cells to cisplatin by cyclopentenylcytosine, in vitro and in vivo. *Life Sciences*, *68*(1), 1–11.

52. Greer, R. M., Peyton, M., Larsen, J. E., Girard, L., Xie, Y., Gazdar, A. F., … Minna, J. D. (2011). SMAC mimetic (JP1201) sensitizes non-small cell lung cancers to multiple chemotherapy agents in an IAP-dependent but TNF-alpha-independent manner. *Cancer Research*, *71*(24), 7640–7648. http://doi.org/10.1158/0008-5472.CAN-10-3947

53. Guo, Y., Chekaluk, Y., Zhang, J., Du, J., Gray, N. S., Wu, C.-L., & Kwiatkowski, D. J. (2013). TSC1 involvement in bladder cancer: diverse effects and therapeutic implications. *The Journal of Pathology*, *230*(1), 17–27. http://doi.org/10.1002/path.4176

54. Gupta, A. K., Cerniglia, G. J., Mick, R., Ahmed, M. S., Bakanauskas, V. J., Muschel, R. J., & McKenna, W. G. (2003). Radiation sensitization of human cancer cells in vivo by inhibiting the activity of PI3K using LY294002. *International Journal of Radiation Oncology, Biology, Physics*, *56*(3), 846–53. Retrieved from http://www.ncbi.nlm.nih.gov/pubmed/12788194

55. Hadaschik, B. A., ter Borg, M. G., Jackson, J., Sowery, R. D., So, A. I., Burt, H. M., & Gleave, M. E. (2008). Paclitaxel and cisplatin as intravesical agents against non-muscle-invasive

bladder cancer. *BJU International*, *101*(11), 1347–1355. http://doi.org/10.1111/j.1464-410X.2008.07571.x

56. Hanada, M., Noguchi, T., & Yamaoka, T. (2007). Amrubicin, a novel 9-aminoanthracycline, enhances the antitumor activity of chemotherapeutic agents against human cancer cells in vitro and in vivo. *Cancer Science*, *98*(3), 447–454. http://doi.org/10.1111/j.1349-7006.2007.00404.x

57. Hanif, F., Perveen, K., Jawed, H., Ahmed, A., Malhi, S. M., Jamall, S., & Simjee, S. U. (2014). N-(2-hydroxyphenyl)acetamide (NA-2) and Temozolomide synergistically induce apoptosis in human glioblastoma cell line U87. *Cancer Cell International*, *14*(1), 133. http://doi.org/10.1186/s12935-014-0133-5

58. Hao, J., Li, Q., Xu, S., Shen, Y., & Sun, G. (2008). Effect of lumiracoxib on proliferation and apoptosis of human nonsmall cell lung cancer cells in vitro. *Chinese Medical Journal*, *121*(7), 602–607.

59. Harris, J. C., Gilliam, A. D., McKenzie, A. J., Evans, S. A., Grabowska, A. M., Clarke, P. A., … Watson, S. A. (2004). The biological and therapeutic importance of gastrin gene expression in pancreatic adenocarcinomas. *Cancer Research*, *64*(16), 5624–5631. http://doi.org/10.1158/0008-5472.CAN-04-0106

60. Hasenstein, J. R., Shin, H.-C., Kasmerchak, K., Buehler, D., Kwon, G. S., & Kozak, K. R. (2012). Antitumor activity of Triolimus: a novel multidrug-loaded micelle containing Paclitaxel, Rapamycin, and 17-AAG. *Molecular Cancer Therapeutics*, *11*(10), 2233–2242. http://doi.org/10.1158/1535-7163.MCT-11-0987

61. Hassan, R., Broaddus, V. C., Wilson, S., Liewehr, D. J., & Zhang, J. (2007). Anti-mesothelin immunotoxin SS1P in combination with gemcitabine results in increased activity against

mesothelin-expressing tumor xenografts. *Clinical Cancer Research : An Official Journal of the American Association for Cancer Research*, *13*(23), 7166–7171. http://doi.org/10.1158/1078-0432.CCR-07-1592

62. Hastak, K., Alli, E., & Ford, J. M. (2010). Synergistic chemosensitivity of triple-negative breast cancer cell lines to poly(ADP-Ribose) polymerase inhibition, gemcitabine, and cisplatin. *Cancer Research*, *70*(20), 7970–7980. http://doi.org/10.1158/0008-5472.CAN-09-4521

63. Haugland, H. K., Nygaard, S. J., & Tysnes, O. B. (1999). Combined effect of alkyl-lysophospholipid and vincristine on proliferation, migration and invasion in human glioma cell lines in vitro. *Anticancer Research*, *19*(1A), 149–156.

64. He, R. Y., & Breitman, T. R. (1991). Retinoic acid inhibits sodium butyrate-induced monocytic differentiation of HL60 cells while synergistically inducing granulocytoid differentiation. *European Journal of Haematology*, *46*(2), 93–100.

65. Hermann, T. W., Yen, W.-C., Tooker, P., Fan, B., Roegner, K., Negro-Vilar, A., … Bissonnette, R. P. (2005). The retinoid X receptor agonist bexarotene (Targretin) synergistically enhances the growth inhibitory activity of cytotoxic drugs in non-small cell lung cancer cells. *Lung Cancer (Amsterdam, Netherlands)*, *50*(1), 9–18. http://doi.org/10.1016/j.lungcan.2005.05.008

66. Hermisson, M., Klumpp, A., Wick, W., Wischhusen, J., Nagel, G., Roos, W., … Weller, M. (2006). O6-methylguanine DNA methyltransferase and p53 status predict temozolomide sensitivity in human malignant glioma cells. *Journal of Neurochemistry*, *96*(3), 766–776. http://doi.org/10.1111/j.1471-4159.2005.03583.x

67. Hirai, H., Sootome, H., Nakatsuru, Y., Miyama, K., Taguchi, S., Tsujioka, K., … Kotani, H. (2010). MK-2206, an allosteric Akt inhibitor, enhances antitumor efficacy by standard chemotherapeutic agents or molecular targeted drugs in vitro and in vivo. *Molecular Cancer Therapeutics*, *9*(7), 1956–1967. http://doi.org/10.1158/1535-7163.MCT-09-1012

68. Hoeflich, K. P., O'Brien, C., Boyd, Z., Cavet, G., Guerrero, S., Jung, K., … Lackner, M. R. (2009). In vivo antitumor activity of MEK and phosphatidylinositol 3-kinase inhibitors in basal-like breast cancer models. *Clinical Cancer Research : An Official Journal of the American Association for Cancer Research*, *15*(14), 4649–4664. http://doi.org/10.1158/1078-0432.CCR-09-0317

69. Honore, S., Kamath, K., Braguer, D., Horwitz, S. B., Wilson, L., Briand, C., & Jordan, M. A. (2004). Synergistic suppression of microtubule dynamics by discodermolide and paclitaxel in non-small cell lung carcinoma cells. *Cancer Research*, *64*(14), 4957–4964. http://doi.org/10.1158/0008-5472.CAN-04-0693

70. Huang, C., Chen, Y. J., Chen, W.-J., Lin, C.-L., Wei, Y. X., & Huang, H. C. (2015). Combined treatment with chrysin and 1,2,3,4,6-penta-O-galloyl-beta-D-glucose synergistically inhibits LRP6 and Skp2 activation in triple-negative breast cancer and xenografts. *Molecular Carcinogenesis*, *54*(12), 1613–1625. http://doi.org/10.1002/mc.22234

71. Huang, L. Y. L., Lee, Y.-S., Huang, J.-J., Chang, C., Chang, J.-M., Chuang, S.-H., … Lau, J. Y. N. (2014). Characterization of the biological activity of a potent small molecule Hec1 inhibitor TAI-1. *Journal of Experimental & Clinical Cancer Research : CR*, *33*, 6. http://doi.org/10.1186/1756-9966-33-6

72. Huang, Y. T., Cheng, C. C., Lin, T. C., Chiu, T. H., & Lai, P. C. (2014). Therapeutic potential of sepantronium bromide YM155 in gemcitabine-resistant human urothelial carcinoma cells. *Oncology Reports*, *31*(2), 771–780. http://doi.org/10.3892/or.2013.2882

73. Hunakova, L., Gronesova, P., Horvathova, E., Chalupa, I., Cholujova, D., Duraj, J., & Sedlak, J. (2014). Modulation of cisplatin sensitivity in human ovarian carcinoma A2780 and SKOV3 cell lines by sulforaphane. *Toxicology Letters*, *230*(3), 479–486. http://doi.org/10.1016/j.toxlet.2014.08.018

74. Hussein, D., Holt, S. V, Brookes, K. E., Klymenko, T., Adamski, J. K., Hogg, A., … Makin, G. W. J. (2009). Preclinical efficacy of the bioreductive alkylating agent RH1 against paediatric tumours. *British Journal of Cancer*, *101*(1), 55–63. http://doi.org/10.1038/sj.bjc.6605100

75. Ide, H., Kikuchi, E., Hasegawa, M., Hattori, S., Yasumizu, Y., Miyajima, A., & Oya, M. (2013). Therapeutic enhancement of S-1 with CPT-11 through down-regulation of thymidylate synthase in bladder cancer. *Cancer Medicine*, *2*(4), 488–95. http://doi.org/10.1002/cam4.95

76. Isoyama, S., Dan, S., Nishimura, Y., Nakamura, N., Kajiwara, G., Seki, M., … Yamori, T. (2012). Establishment of phosphatidylinositol 3-kinase inhibitor-resistant cancer cell lines and therapeutic strategies for overcoming the resistance. *Cancer Science*, *103*(11), 1955–1960. http://doi.org/10.1111/cas.12004

77. Jacob, E., Scorsone, K., Blaney, S. M., D'Argenio, D. Z., & Berg, S. L. (2008). Synergy of karenitecin and mafosfamide in pediatric leukemia, medulloblastoma, and neuroblastoma cell lines. *Pediatric Blood & Cancer*, *50*(4), 757–760. http://doi.org/10.1002/pbc.21330

78. Janss, A. J., Cnaan, A., Zhao, H., Shpilsky, A., Levow, C., Sutton, L., & Phillips, P. C. (1998). Synergistic cytotoxicity of topoisomerase I inhibitors with alkylating agents and etoposide in human brain tumor cell lines. *Anti-Cancer Drugs*, *9*(7), 641–652.

79. Jaszberenyi, M., Rick, F. G., Popovics, P., Block, N. L., Zarandi, M., Cai, R.-Z., … Schally, A. V. (2014). Potentiation of cytotoxic chemotherapy by growth hormone-releasing hormone agonists. *Proceedings of the National Academy of Sciences of the United States of America*, *111*(2), 781–786. http://doi.org/10.1073/pnas.1322622111

80. Jeon, H. G., Yoon, C. Y., Yu, J. H., Park, M. J., Lee, J. E., Jeong, S. J., … Lee, S. E. (2011). Induction of caspase mediated apoptosis and down-regulation of nuclear factor-kappaB and Akt signaling are involved in the synergistic antitumor effect of gemcitabine and the histone deacetylase inhibitor trichostatin A in human bladder cancer cells. *The Journal of Urology*, *186*(5), 2084–2093. http://doi.org/10.1016/j.juro.2011.06.053

81. Johnston, J. S., Johnson, A., Gan, Y., Wientjes, M. G., & Au, J. L. S. (2003). Synergy between 3’-azido-3’-deoxythymidine and paclitaxel in human pharynx FaDu cells. *Pharmaceutical Research*, *20*(7), 957–961.

82. Jung, J. H., Kwon, T.-R., Jeong, S.-J., Kim, E.-O., Sohn, E. J., Yun, M., & Kim, S.-H. (2013). Apoptosis Induced by Tanshinone IIA and Cryptotanshinone Is Mediated by Distinct JAK/STAT3/5 and SHP1/2 Signaling in Chronic Myeloid Leukemia K562 Cells. *Evidence-Based Complementary and Alternative Medicine : eCAM*, *2013*, 805639. http://doi.org/10.1155/2013/805639

83. Kamat, A. M., DeHaven, J. I., & Lamm, D. L. (1999). Quinolone antibiotics: a potential adjunct to intravesical chemotherapy for bladder cancer. *Urology*, *54*(1), 56–61.

84. Karpel-Massler, G., Westhoff, M.-A., Zhou, S., Nonnenmacher, L., Dwucet, A., Kast, R. E., … Halatsch, M.-E. (2013). Combined inhibition of HER1/EGFR and RAC1 results in a synergistic antiproliferative effect on established and primary cultured human glioblastoma cells. *Molecular Cancer Therapeutics*, *12*(9), 1783–1795. http://doi.org/10.1158/1535-7163.MCT-13-0052

85. Katt, W. P., Antonyak, M. A., & Cerione, R. A. (2015). Simultaneously targeting tissue transglutaminase and kidney type glutaminase sensitizes cancer cells to acid toxicity and offers new opportunities for therapeutic intervention. *Molecular Pharmaceutics*, *12*(1), 46–55. http://doi.org/10.1021/mp500405h

86. Kaur, G., Behrsing, H., Parchment, R. E., Millin, M. D., & Teicher, B. A. (2013). Analyses of the combination of 6-MP and dasatinib in cell culture. *International Journal of Oncology*, *43*(1), 13–22. http://doi.org/10.3892/ijo.2013.1930

87. Kern, D. H., Morgan, C. R., & Hildebrand-Zanki, S. U. (1988). In vitro pharmacodynamics of 1-beta-D-arabinofuranosylcytosine: synergy of antitumor activity with cis-diamminedichloroplatinum(II). *Cancer Research*, *48*(1), 117–121.

88. Kim, J. S., Amorino, G. P., Pyo, H., Cao, Q., Price, J. O., & Choy, H. (2001). The novel taxane analogs, BMS-184476 and BMS-188797, potentiate the effects of radiation therapy in vitro and in vivo against human lung cancer cells. *International Journal of Radiation Oncology, Biology, Physics*, *51*(2), 525–534.

89. Koay, D. C., Zerillo, C., Narayan, M., Harris, L. N., & DiGiovanna, M. P. (2010). Anti-tumor effects of retinoids combined with trastuzumab or tamoxifen in breast cancer cells: induction of apoptosis by retinoid/trastuzumab combinations. *Breast Cancer Research : BCR*, *12*(4), R62. http://doi.org/10.1186/bcr2625

90. Koll, T. T., Feis, S. S., Wright, M. H., Teniola, M. M., Richardson, M. M., Robles, A. I., … Varticovski, L. (2008). HSP90 inhibitor, DMAG, synergizes with radiation of lung cancer cells by interfering with base excision and ATM-mediated DNA repair. *Molecular Cancer Therapeutics*, *7*(7), 1985–1992. http://doi.org/10.1158/1535-7163.MCT-07-2104

91. Kondo, S., Yin, D., Morimura, T., & Takeuchi, J. (1995). Combination therapy with cisplatin and nifedipine inducing apoptosis in multidrug-resistant human glioblastoma cells. *Journal of Neurosurgery*, *82*(3), 469–474. http://doi.org/10.3171/jns.1995.82.3.0469

92. Konecny, G. E., Pegram, M. D., Venkatesan, N., Finn, R., Yang, G., Rahmeh, M., … Slamon, D. J. (2006). Activity of the dual kinase inhibitor lapatinib (GW572016) against HER-2-overexpressing and trastuzumab-treated breast cancer cells. *Cancer Research*, *66*(3), 1630–1639. http://doi.org/10.1158/0008-5472.CAN-05-1182

93. Korelitz, B. I., & Sommers, S. C. (1975). Responses to drug therapy in ulcerative colitis. Evaluation by rectal biopsy and histopathological changes. *The American Journal of Gastroenterology*, *64*(5), 365–370.

94. Koto, K. S., Lescault, P., Brard, L., Kim, K., Singh, R. K., Bond, J., … Saulnier Sholler, G. L. (2011). Antitumor activity of nifurtimox is enhanced with tetrathiomolybdate in medulloblastoma. *International Journal of Oncology*, *38*(5), 1329–1341. http://doi.org/10.3892/ijo.2011.971

95. Kouame, P. B.-K., Jacques, C., Bedi, G., Silvestre, V., Loquet, D., Barille-Nion, S., … Tea, I. (2013). Phytochemicals isolated from leaves of Chromolaena odorata: impact on viability and clonogenicity of cancer cell lines. *Phytotherapy Research : PTR*, *27*(6), 835–840. http://doi.org/10.1002/ptr.4787

96. Ku, B. M., Jho, E. H., Bae, Y.-H., Sun, J.-M., Ahn, J. S., Park, K., & Ahn, M.-J. (2015). BYL719, a selective inhibitor of phosphoinositide 3-Kinase alpha, enhances the effect of selumetinib (AZD6244, ARRY-142886) in KRAS-mutant non-small cell lung cancer. *Investigational New Drugs*, *33*(1), 12–21. http://doi.org/10.1007/s10637-014-0163-9

97. Kwok, C. W., Treeck, O., Buchholz, S., Seitz, S., Ortmann, O., & Engel, J. B. (2015). Receptors for luteinizing hormone-releasing hormone (GnRH) as therapeutic targets in triple negative breast cancers (TNBC). *Targeted Oncology*, *10*(3), 365–373. http://doi.org/10.1007/s11523-014-0340-y

98. Lau, Y.-K. I., Du, X., Rayannavar, V., Hopkins, B., Shaw, J., Bessler, E., … Maurer, M. A. (2014). Metformin and erlotinib synergize to inhibit basal breast cancer. *Oncotarget*, *5*(21), 10503–10517. http://doi.org/10.18632/oncotarget.2391

99. Lazaro, G., Smith, C., Goddard, L., Jordan, N., McClelland, R., Barrett-Lee, P., … Hiscox, S. (2013). Targeting focal adhesion kinase in ER+/HER2+ breast cancer improves trastuzumab response. *Endocrine-Related Cancer*, *20*(5), 691–704. http://doi.org/10.1530/ERC-13-0019

100. Lee, Y. K., Isham, C. R., Kaufman, S. H., & Bible, K. C. (2006). Flavopiridol disrupts STAT3/DNA interactions, attenuates STAT3-directed transcription, and combines with the Jak kinase inhibitor AG490 to achieve cytotoxic synergy. *Molecular Cancer Therapeutics*, *5*(1), 138–148. http://doi.org/10.1158/1535-7163.MCT-05-0235

101. Lenzi, R., Frost, P., & Abbruzzese, J. L. (1994). Modulation of cisplatin resistance by 2’-deoxy-5-azacytidine in human ovarian tumor cell lines. *Anticancer Research*, *14*(1A), 247–251.

102. Leung, E. Y., Kim, J. E., Askarian-Amiri, M., Rewcastle, G. W., Finlay, G. J., & Baguley, B. C. (2014). Relationships between signaling pathway usage and sensitivity to a pathway inhibitor: examination of trametinib responses in cultured breast cancer lines. *PloS One*, *9*(8), e105792. http://doi.org/10.1371/journal.pone.0105792

103. Li, G., Miskimen, K. L., Wang, Z., Xie, X. Y., Tse, W., Gouilleux, F., … Bunting, K. D. (2010). Effective targeting of STAT5-mediated survival in myeloproliferative neoplasms using ABT-737 combined with rapamycin. *Leukemia*, *24*(8), 1397–1405. http://doi.org/10.1038/leu.2010.131

104. Li, J., Pan, Y.-Y., & Zhang, Y. (2013). Sorafenib combined with gemcitabine in EGFR-TKI-resistant human lung cancer cells. *Oncology Letters*, *5*(1), 68–72. http://doi.org/10.3892/ol.2012.958

105. Li, L., Keating, M. J., Plunkett, W., & Yang, L. Y. (1997). Fludarabine-mediated repair inhibition of cisplatin-induced DNA lesions in human chronic myelogenous leukemia-blast crisis K562 cells: induction of synergistic cytotoxicity independent of reversal of apoptosis resistance. *Molecular Pharmacology*, *52*(5), 798–806.

106. Li, Y., Yang, X., Su, L.-J., & Flaig, T. W. (2010). VEGFR and EGFR inhibition increases epithelial cellular characteristics and chemotherapy sensitivity in mesenchymal bladder cancer cells. *Oncology Reports*, *24*(4), 1019–28. Retrieved from http://www.ncbi.nlm.nih.gov/pubmed/20811684

107. Li, Y., Yang, X., Su, L.-J., & Flaig, T. W. (2011). Pazopanib synergizes with docetaxel in the treatment of bladder cancer cells. *Urology*, *78*(1), 233.e7-13. http://doi.org/10.1016/j.urology.2011.02.041

108. Liang, H., Wang, H. B., Liu, H. Z., Wen, X. J., Zhou, Q. L., & Yang, C. X. (2013). The effects of combined treatment with sevoflurane and cisplatin on growth and invasion of human adenocarcinoma cell line A549. *Biomedicine & Pharmacotherapy = Biomedecine & Pharmacotherapie*, *67*(6), 503–509. http://doi.org/10.1016/j.biopha.2013.03.005

109. Lin, T., Meng, L., & Tsai, R. Y. L. (2011). GTP depletion synergizes the anti-proliferative activity of chemotherapeutic agents in a cell type-dependent manner. *Biochemical and Biophysical Research Communications*, *414*(2), 403–408. http://doi.org/10.1016/j.bbrc.2011.09.091

110. Lirk, P., Hollmann, M. W., Fleischer, M., Weber, N. C., & Fiegl, H. (2014). Lidocaine and ropivacaine, but not bupivacaine, demethylate deoxyribonucleic acid in breast cancer cells in vitro. *British Journal of Anaesthesia*, *113 Suppl*, i32-8. http://doi.org/10.1093/bja/aeu201

111. Liu, D., Xing, J., Trink, B., & Xing, M. (2010). BRAF mutation-selective inhibition of thyroid cancer cells by the novel MEK inhibitor RDEA119 and genetic-potentiated synergism with the mTOR inhibitor temsirolimus. *International Journal of Cancer*, *127*(12), 2965–2973. http://doi.org/10.1002/ijc.25304

112. Liu, H., Scholz, C., Zang, C., Schefe, J. H., Habbel, P., Regierer, A.-C., … Eucker, J. (2012). Metformin and the mTOR inhibitor everolimus (RAD001) sensitize breast cancer cells to the cytotoxic effect of chemotherapeutic drugs in vitro. *Anticancer Research*, *32*(5), 1627–1637.

113. Liu, H., Zang, C., Schefe, J.-H., Schwarzlose-Schwarck, S., Regierer, A.-C., Elstner, E., … Eucker, J. (2011). The mTOR inhibitor RAD001 sensitizes tumor cells to the cytotoxic effect of carboplatin in breast cancer in vitro. *Anticancer Research*, *31*(9), 2713–2722.

114. Liu, L., Shi, H., Liu, Y., Anderson, A., Peterson, J., Greger, J., … Gilmer, T. M. (2011). Synergistic effects of foretinib with HER-targeted agents in MET and HER1- or HER2-coactivated tumor cells. *Molecular Cancer Therapeutics*, *10*(3), 518–530. http://doi.org/10.1158/1535-7163.MCT-10-0698

115. Liu, P., Xu, S., Zhang, M., Wang, W. W., Zhang, Y. F., Rehman, K., … Chen, Z. (2013). Anticancer activity in human multiple myeloma U266 cells: synergy between cryptotanshinone and arsenic trioxide. *Metallomics : Integrated Biometal Science*, *5*(7), 871–878. http://doi.org/10.1039/c3mt20272k

116. Liu, S., Monks, N. R., Hanes, J. W., Begley, T. P., Yu, H., & Moscow, J. A. (2010). Sensitivity of breast cancer cell lines to recombinant thiaminase I. *Cancer Chemotherapy and Pharmacology*, *66*(1), 171–179. http://doi.org/10.1007/s00280-009-1148-9

117. Liu, W., Xu, J., Liu, Y., Yu, X., Tang, X., Wang, Z., & Li, X. (2014). Anthocyanins potentiate the activity of trastuzumab in human epidermal growth factor receptor 2-positive breast cancer cells in vitro and in vivo. *Molecular Medicine Reports*, *10*(4), 1921–1926. http://doi.org/10.3892/mmr.2014.2414

118. Lu, S., Chen, Z., Yang, J., Chen, L., Zhou, H., Xu, X., … Wang, J. (2010). The effects of proteasome inhibitor bortezomib on a P-gp positive leukemia cell line K562/A02. *International Journal of Laboratory Hematology*, *32*(1 Pt 1), e123-31. http://doi.org/10.1111/j.1751-553X.2009.01145.x

119. Lu, X.-Y., Cao, K., Li, Q.-Y., Yuan, Z.-C., & Lu, P.-S. (2012). The synergistic therapeutic effect of temozolomide and hyperbaric oxygen on glioma U251 cell lines is accompanied by alterations in vascular endothelial growth factor and multidrug resistance-associated protein-1 levels. *The Journal of International Medical Research*, *40*(3), 995–1004.

120. Ma, C., Niu, X., Luo, J., Shao, Z., & Shen, K. (2010). Combined effects of lapatinib and bortezomib in human epidermal receptor 2 (HER2)-overexpressing breast cancer cells and activity of bortezomib against lapatinib-resistant breast cancer cells. *Cancer Science*, *101*(10), 2220–2226. http://doi.org/10.1111/j.1349-7006.2010.01662.x

121. Makar, A. B., McMartin, K. E., Palese, M., & Tephly, T. R. (1975). Formate assay in body fluids: application in methanol poisoning. *Biochemical Medicine*, *13*(2), 117–126.

122. Maneksha, S., & Harry, T. V. (1975). Lorazepam in sexual disorders. *The British Journal of Clinical Practice*, *29*(7), 175–176.

123. Marcu, J. P., Christian, R. T., Lau, D., Zielinski, A. J., Horowitz, M. P., Lee, J., … McAllister, S. D. (2010). Cannabidiol enhances the inhibitory effects of delta9-tetrahydrocannabinol on human glioblastoma cell proliferation and survival. *Molecular Cancer Therapeutics*, *9*(1), 180–189. http://doi.org/10.1158/1535-7163.MCT-09-0407

124. Martello, L. A., McDaid, H. M., Regl, D. L., Yang, C. P., Meng, D., Pettus, T. R., … Horwitz, S. B. (2000). Taxol and discodermolide represent a synergistic drug combination in human carcinoma cell lines. *Clinical Cancer Research : An Official Journal of the American Association for Cancer Research*, *6*(5), 1978–1987.

125. Martin, A. P., Mitchell, C., Rahmani, M., Nephew, K. P., Grant, S., & Dent, P. (2009). Inhibition of MCL-1 enhances lapatinib toxicity and overcomes lapatinib resistance via BAK-dependent autophagy. *Cancer Biology & Therapy*, *8*(21), 2084–2096.

126. Matar, P., Rojo, F., Cassia, R., Moreno-Bueno, G., Di Cosimo, S., Tabernero, J., … Baselga, J. (2004). Combined epidermal growth factor receptor targeting with the tyrosine kinase inhibitor gefitinib (ZD1839) and the monoclonal antibody cetuximab (IMC-C225): superiority over single-agent receptor targeting. *Clinical Cancer Research : An Official*

Journal of the American Association for Cancer Research, *10*(19), 6487–6501. http://doi.org/10.1158/1078-0432.CCR-04-0870

127. Mathew, M. P., Tan, E., Saeui, C. T., Bovonratwet, P., Liu, L., Bhattacharya, R., & Yarema, K. J. (2015). Metabolic glycoengineering sensitizes drug-resistant pancreatic cancer cells to tyrosine kinase inhibitors erlotinib and gefitinib. *Bioorganic & Medicinal Chemistry Letters*, *25*(6), 1223–1227. http://doi.org/10.1016/j.bmcl.2015.01.060

128. Matsui, Y., Watanabe, J., Ding, S., Nishizawa, K., Kajita, Y., Ichioka, K., … Nishiyama, H. (2010). Dicoumarol enhances doxorubicin-induced cytotoxicity in p53 wild-type urothelial cancer cells through p38 activation. *BJU International*, *105*(4), 558–564. http://doi.org/10.1111/j.1464-410X.2009.08732.x

129. Matsumoto, M., Kawahara, T., Tsuda, M., Ohkubo, T., Kamiguchi, H., & Yamamura, M. (1992). Synergistic inhibition of human gastric carcinoma cell growth by 1-beta-D-arabinofuranosylcytosine and hydroxyurea or 2’-deoxyguanosine in vitro. *Cancer Letters*, *63*(3), 221–228.

130. McPherson, R. A. C., Galettis, P. T., & de Souza, P. L. (2009). Enhancement of the activity of phenoxodiol by cisplatin in prostate cancer cells. *British Journal of Cancer*, *100*(4), 649–655. http://doi.org/10.1038/sj.bjc.6604920

131. Menendez, J. A., Vellon, L., Colomer, R., & Lupu, R. (2005). Effect of gamma-linolenic acid on the transcriptional activity of the Her-2/neu (erbB-2) oncogene. *Journal of the National Cancer Institute*, *97*(21), 1611–1615. http://doi.org/10.1093/jnci/dji343

132. Menendez, J. A., Vellon, L., Colomer, R., & Lupu, R. (2005). Oleic acid, the main monounsaturated fatty acid of olive oil, suppresses Her-2/neu (erbB-2) expression and synergistically enhances the growth inhibitory effects of trastuzumab (Herceptin) in breast

cancer cells with Her-2/neu oncogene amplification. *Annals of Oncology : Official Journal of the European Society for Medical Oncology / ESMO*, *16*(3), 359–371. http://doi.org/10.1093/annonc/mdi090

133. Meng, J., Dai, B., Fang, B., Bekele, B. N., Bornmann, W. G., Sun, D., … Roth, J. A. (2010). Combination treatment with MEK and AKT inhibitors is more effective than each drug alone in human non-small cell lung cancer in vitro and in vivo. *PloS One*, *5*(11), e14124. http://doi.org/10.1371/journal.pone.0014124

134. Mey, V., Giovannetti, E., De Braud, F., Nannizzi, S., Curigliano, G., Verweij, F., … Danesi, R. (2006). In vitro synergistic cytotoxicity of gemcitabine and pemetrexed and pharmacogenetic evaluation of response to gemcitabine in bladder cancer patients. *British Journal of Cancer*, *95*(3), 289–297. http://doi.org/10.1038/sj.bjc.6603242

135. Miki, K., Al-Refaie, W., Xu, M., Jiang, P., Tan, Y., Bouvet, M., … Hoffman, R. M. (2000). Methioninase gene therapy of human cancer cells is synergistic with recombinant methioninase treatment. *Cancer Research*, *60*(10), 2696–2702.

136. Milella, M., Trisciuoglio, D., Bruno, T., Ciuffreda, L., Mottolese, M., Cianciulli, A., … Zupi, G. (2004). Trastuzumab down-regulates Bcl-2 expression and potentiates apoptosis induction by Bcl-2/Bcl-XL bispecific antisense oligonucleotides in HER-2 gene--amplified breast cancer cells. *Clinical Cancer Research : An Official Journal of the American Association for Cancer Research*, *10*(22), 7747–7756. http://doi.org/10.1158/1078-0432.CCR-04-0908

137. Mitchell, C., Yacoub, A., Hossein, H., Martin, A. P., Bareford, M. D., Eulitt, P., … Dent, P. (2010). Inhibition of MCL-1 in breast cancer cells promotes cell death in vitro and in vivo. *Cancer Biology & Therapy*, *10*(9), 903–917. http://doi.org/10.4161/cbt.10.9.13273

138. Mittendorf, E. A., Liu, Y., Tucker, S. L., McKenzie, T., Qiao, N., Akli, S., … Hunt, K. K. (2010). A novel interaction between HER2/neu and cyclin E in breast cancer. *Oncogene*, *29*(27), 3896–3907. http://doi.org/10.1038/onc.2010.151

139. Mizutani, Y., Kamoi, K., Ukimura, O., Kawauchi, A., & Miki, T. (2002). Synergistic cytotoxicity and apoptosis of JTE-522, a selective cyclooxygenase-2 inhibitor, and 5-fluorouracil against bladder cancer. *The Journal of Urology*, *168*(6), 2650–2654. http://doi.org/10.1097/01.ju.0000030150.25914.c6

140. Mizutani, Y., Nakanishi, H., Li, Y. N., Sato, N., Kawauchi, A., & Miki, T. (2004). Enhanced sensitivity of bladder cancer cells to cisplatin mediated cytotoxicity and apoptosis in vitro and in vivo by the selective cyclooxygenase-2 inhibitor JTE-522. *The Journal of Urology*, *172*(4 Pt 1), 1474–1479.

141. Mizutani, Y., Okada, Y., Yoshida, O., Fukumoto, M., & Bonavida, B. (1997). Doxorubicin sensitizes human bladder carcinoma cells to Fas-mediated cytotoxicity. *Cancer*, *79*(6), 1180–1189.

142. Mizutani, Y., Wu, X. X., Yoshida, O., Shirasaka, T., & Bonavida, B. (1999). Chemoimmunosensitization of the T24 human bladder cancer line to Fas-mediated cytotoxicity and apoptosis by cisplatin and 5-fluorouracil. *Oncology Reports*, *6*(5), 979–982.

143. Mizutani, Y., Yoshida, O., & Bonavida, B. (1998). Sensitization of human bladder cancer cells to Fas-mediated cytotoxicity by cis-diamminedichloroplatinum (II). *The Journal of Urology*, *160*(2), 561–570.

144. Montero, A. J., Diaz-Montero, C. M., Mao, L., Youssef, E. M., Estecio, M., Shen, L., & Issa, J.-P. J. (2006). Epigenetic inactivation of EGFR by CpG island hypermethylation in cancer. *Cancer Biology & Therapy*, *5*(11), 1494–1501.

145. Moran, R. G., & Scanlon, K. L. (1991). Schedule-dependent enhancement of the cytotoxicity of fluoropyrimidines to human carcinoma cells in the presence of folinic acid. *Cancer Research*, *51*(17), 4618–4623.

146. Moufarij, M. A., Phillips, D. R., & Cullinane, C. (2003). Gemcitabine potentiates cisplatin cytotoxicity and inhibits repair of cisplatin-DNA damage in ovarian cancer cell lines. *Molecular Pharmacology*, *63*(4), 862–869.

147. Munster, P. N., Basso, A., Solit, D., Norton, L., & Rosen, N. (2001). Modulation of Hsp90 function by ansamycins sensitizes breast cancer cells to chemotherapy-induced apoptosis in an RB- and schedule-dependent manner. See: E. A. Sausville, Combining cytotoxics and 17-allylamino, 17-demethoxygeldanamycin: sequence and tumor biology matters, Clin. Cancer Res., 7: 2155-2158, 2001. *Clinical Cancer Research : An Official Journal of the American Association for Cancer Research*, *7*(8), 2228–2236.

148. Murai, J., Zhang, Y., Morris, J., Ji, J., Takeda, S., Doroshow, J. H., & Pommier, Y. (2014). Rationale for poly(ADP-ribose) polymerase (PARP) inhibitors in combination therapy with camptothecins or temozolomide based on PARP trapping versus catalytic inhibition. *The Journal of Pharmacology and Experimental Therapeutics*, *349*(3), 408–416. http://doi.org/10.1124/jpet.113.210146

149. Naderi, A., Chia, K. M., & Liu, J. (2011). Synergy between inhibitors of androgen receptor and MEK has therapeutic implications in estrogen receptor-negative breast cancer. *Breast Cancer Research : BCR*, *13*(2), R36. http://doi.org/10.1186/bcr2858

150. Nagaria, T. S., Williams, J. L., Leduc, C., Squire, J. A., Greer, P. A., & Sangrar, W. (2013). Flavopiridol synergizes with sorafenib to induce cytotoxicity and potentiate antitumorigenic activity in EGFR/HER-2 and mutant RAS/RAF breast cancer model systems. *Neoplasia (New York, N.Y.)*, *15*(8), 939–951.

151. Nahta, R., Hung, M.-C., & Esteva, F. J. (2004). The HER-2-targeting antibodies trastuzumab and pertuzumab synergistically inhibit the survival of breast cancer cells. *Cancer Research*, *64*(7), 2343–2346.

152. Nahta, R., Iglehart, J. D., Kempkes, B., & Schmidt, E. V. (2002). Rate-limiting effects of Cyclin D1 in transformation by ErbB2 predicts synergy between herceptin and flavopiridol. *Cancer Research*, *62*(8), 2267–2271.

153. Nahta, R., Trent, S., Yang, C., & Schmidt, E. V. (2003). Epidermal growth factor receptor expression is a candidate target of the synergistic combination of trastuzumab and flavopiridol in breast cancer. *Cancer Research*, *63*(13), 3626–3631.

154. Nautiyal, J., Yu, Y., Aboukameel, A., Kanwar, S. S., Das, J. K., Du, J., … Majumdar, A. P. N. (2010). ErbB-inhibitory protein: a modified ectodomain of epidermal growth factor receptor synergizes with dasatinib to inhibit growth of breast cancer cells. *Molecular Cancer Therapeutics*, *9*(6), 1503–1514. http://doi.org/10.1158/1535-7163.MCT-10-0019

155. Normanno, N., Campiglio, M., De, L. A., Somenzi, G., Maiello, M., Ciardiello, F., … Menard, S. (2002). Cooperative inhibitory effect of ZD1839 (Iressa) in combination with trastuzumab (Herceptin) on human breast cancer cell growth. *Annals of Oncology : Official Journal of the European Society for Medical Oncology / ESMO*, *13*(1), 65–72.

156. Nugroho, A. E., Hermawan, A., Putri D, P., Meiyanto, E., & Hakim, L. (2012). Synergistic effects of ethyl acetate fraction of Ficus septica Burm. f. and doxorubicin chemotherapy

on T47D human breast cancer cell line. *Zhong Xi Yi Jie He Xue Bao = Journal of Chinese Integrative Medicine*, *10*(10), 1162–1170.

157. O'Donnell, R. T., DeNardo, S. J., Miers, L. A., Kukis, D. L., Mirick, G. R., Kroger, L. A., & DeNardo, G. L. (1998). Combined modality radioimmunotherapy with Taxol and 90Y-Lym-1 for Raji lymphoma xenografts. *Cancer Biotherapy & Radiopharmaceuticals*, *13*(5), 351–361. http://doi.org/10.1089/cbr.1998.13.351

158. Oliver, P. G., LoBuglio, A. F., Zhou, T., Forero, A., Kim, H., Zinn, K. R., … Buchsbaum, D. J. (2012). Effect of anti-DR5 and chemotherapy on basal-like breast cancer. *Breast Cancer Research and Treatment*, *133*(2), 417–426. http://doi.org/10.1007/s10549-011-1755-0

159. Oyaizu, H., Adachi, Y., Okumura, T., Okigaki, M., Oyaizu, N., Taketani, S., … Ikehara, S. (2001). Proteasome inhibitor 1 enhances paclitaxel-induced apoptosis in human lung adenocarcinoma cell line. *Oncology Reports*, *8*(4), 825–829.

160. Ozawa, Y., Kusano, K., Owa, T., Yokoi, A., Asada, M., & Yoshimatsu, K. (2012). Therapeutic potential and molecular mechanism of a novel sulfonamide anticancer drug, indisulam (E7070) in combination with CPT-11 for cancer treatment. *Cancer Chemotherapy and Pharmacology*, *69*(5), 1353–1362. http://doi.org/10.1007/s00280-012-1844-8

161. Paller, C. J., Wissing, M. D., Mendonca, J., Sharma, A., Kim, E., Kim, H.-S., … Kachhap, S. K. (2014). Combining the pan-aurora kinase inhibitor AMG 900 with histone deacetylase inhibitors enhances antitumor activity in prostate cancer. *Cancer Medicine*, *3*(5), 1322–1335. http://doi.org/10.1002/cam4.289.

162. Park JH, Koo SY, Dong-Myung Kim, Kim K, Jeong SW, Chung HH, Heung-Soo Cho, Park J, Yim HJ, Lee J, Koh JS, and Kim S. Antitumor Activity of LB42907, a Potent and Selective

Farnesyltransferase Inhibitor: Synergistic Effect in Combination with Other Anticancer Drugs. Bull. Korean Chem. Soc. 2008, Vol. 29, No. 7 1303.

163. Pegram, M. D., Konecny, G. E., O'Callaghan, C., Beryt, M., Pietras, R., & Slamon, D. J. (2004). Rational combinations of trastuzumab with chemotherapeutic drugs used in the treatment of breast cancer. *Journal of the National Cancer Institute*, *96*(10), 739–749.

164. Perez, R. P., Perez, K. M., Handel, L. M., & Hamilton, T. C. (1992). In vitro interactions between platinum analogues in human ovarian-carcinoma cell lines. *Cancer Chemotherapy and Pharmacology*, *29*(6), 430–434.

165. Peters, G. J., Bergman, A. M., Ruiz van Haperen, V. W., Veerman, G., Kuiper, C. M., & Braakhuis, B. J. (1995). Interaction between cisplatin and gemcitabine in vitro and in vivo. *Seminars in Oncology*, *22*(4 Suppl 11), 72–79.

166. Phillips, G. D. L., Fields, C. T., Li, G., Dowbenko, D., Schaefer, G., Miller, K., … Sliwkowski, M. X. (2014). Dual targeting of HER2-positive cancer with trastuzumab emtansine and pertuzumab: critical role for neuregulin blockade in antitumor response to combination therapy. *Clinical Cancer Research : An Official Journal of the American Association for Cancer Research*, *20*(2), 456–468. http://doi.org/10.1158/1078-0432.CCR-13-0358

167. Photiou, A., Shah, P., Leong, L. K., Moss, J., & Retsas, S. (1997). In vitro synergy of paclitaxel (Taxol) and vinorelbine (navelbine) against human melanoma cell lines. *European Journal of Cancer (Oxford, England : 1990)*, *33*(3), 463–470.

168. Pichot, C. S., Hartig, S. M., Xia, L., Arvanitis, C., Monisvais, D., Lee, F. Y., … Corey, S. J. (2009). Dasatinib synergizes with doxorubicin to block growth, migration, and invasion of breast cancer cells. *British Journal of Cancer*, *101*(1), 38–47. http://doi.org/10.1038/sj.bjc.6605101

169. Pledgie-Tracy, A., Billam, M., Hacker, A., Sobolewski, M. D., Woster, P. M., Zhang, Z., … Davidson, N. E. (2010). The role of the polyamine catabolic enzymes SSAT and SMO in the synergistic effects of standard chemotherapeutic agents with a polyamine analogue in human breast cancer cell lines. *Cancer Chemotherapy and Pharmacology*, *65*(6), 1067–1081. http://doi.org/10.1007/s00280-009-1112-8

170. Plowman, J., Waud, W. R., Koutsoukos, A. D., Rubinstein, L. V, Moore, T. D., & Grever, M. R. (1994). Preclinical antitumor activity of temozolomide in mice: efficacy against human brain tumor xenografts and synergism with 1,3-bis(2-chloroethyl)-1-nitrosourea. *Cancer Research*, *54*(14), 3793–3799.

171. Potuckova, E., Jansova, H., Machacek, M., Vavrova, A., Haskova, P., Tichotova, L., … Simunek, T. (2014). Quantitative analysis of the anti-proliferative activity of combinations of selected iron-chelating agents and clinically used anti-neoplastic drugs. *PloS One*, *9*(2), e88754. http://doi.org/10.1371/journal.pone.0088754

172. Pozdeyev, N., Berlinberg, A., Zhou, Q., Wuensch, K., Shibata, H., Wood, W. M., & Haugen, B. R. (2015). Targeting the NF-kappaB Pathway as a Combination Therapy for Advanced Thyroid Cancer. *PloS One*, *10*(8), e0134901. http://doi.org/10.1371/journal.pone.0134901

173. Puig, T., Aguilar, H., Cufi, S., Oliveras, G., Turrado, C., Ortega-Gutierrez, S., … Colomer, R. (2011). A novel inhibitor of fatty acid synthase shows activity against HER2+ breast cancer xenografts and is active in anti-HER2 drug-resistant cell lines. *Breast Cancer Research : BCR*, *13*(6), R131. http://doi.org/10.1186/bcr3077

174. Qi, Y., Fu, X., Xiong, Z., Zhang, H., Hill, S. M., Rowan, B. G., & Dong, Y. (2012). Methylseleninic acid enhances paclitaxel efficacy for the treatment of triple-negative breast cancer. *PloS One*, *7*(2), e31539. http://doi.org/10.1371/journal.pone.0031539

175. Qiu, Z.-K., Shen, D., Chen, Y.-S., Yang, Q.-Y., Guo, C.-C., Feng, B.-H., & Chen, Z.-P. (2014). Enhanced MGMT expression contributes to temozolomide resistance in glioma stem-like cells. *Chinese Journal of Cancer*, *33*(2), 115–122. http://doi.org/10.5732/cjc.012.10236

176. Raina, D., Uchida, Y., Kharbanda, A., Rajabi, H., Panchamoorthy, G., Jin, C., … Kufe, D. (2014). Targeting the MUC1-C oncoprotein downregulates HER2 activation and abrogates trastuzumab resistance in breast cancer cells. *Oncogene*, *33*(26), 3422–3431. http://doi.org/10.1038/onc.2013.308

177. Raja, S. M., Clubb, R. J., Bhattacharyya, M., Dimri, M., Cheng, H., Pan, W., … Band, H. (2008). A combination of Trastuzumab and 17-AAG induces enhanced ubiquitinylation and lysosomal pathway-dependent ErbB2 degradation and cytotoxicity in ErbB2-overexpressing breast cancer cells. *Cancer Biology & Therapy*, *7*(10), 1630–1640.

178. Robinson, J. P., Vanbrocklin, M. W., Lastwika, K. J., McKinney, A. J., Brandner, S., & Holmen, S. L. (2011). Activated MEK cooperates with Ink4a/Arf loss or Akt activation to induce gliomas in vivo. *Oncogene*, *30*(11), 1341–1350. http://doi.org/10.1038/onc.2010.513

179. Rogers, M., Kalra, S., Moukharskaya, J., Chakraborty, K., Niyazi, M., Krishnan, K., … Palau, V. E. (2015). Synergistic growth inhibition of PC3 prostate cancer cells with low-dose combinations of simvastatin and alendronate. *Anticancer Research*, *35*(4), 1851–1859.

180. Rose, P. G. (2005). Gemcitabine reverses platinum resistance in platinum-resistant ovarian and peritoneal carcinoma. *International Journal of Gynecological Cancer : Official Journal of the International Gynecological Cancer Society*, *15 Suppl 1*, 18–22. http://doi.org/10.1111/j.1525-1438.2005.15357.x

181. Rose, P. G., Mossbruger, K., Fusco, N., Smrekar, M., Eaton, S., & Rodriguez, M. (2003). Gemcitabine reverses cisplatin resistance: demonstration of activity in platinum- and

multidrug-resistant ovarian and peritoneal carcinoma. *Gynecologic Oncology*, *88*(1), 17–21.

182. Rosik, L., Niegisch, G., Fischer, U., Jung, M., Schulz, W. A., & Hoffmann, M. J. (2014). Limited efficacy of specific HDAC6 inhibition in urothelial cancer cells. *Cancer Biology & Therapy*, *15*(6), 742–757. http://doi.org/10.4161/cbt.28469
183. Rubel, A., Handrick, R., Lindner, L. H., Steiger, M., Eibl, H., Budach, W., … Jendrossek, V. (2006). The membrane targeted apoptosis modulators erucylphosphocholine and erucylphosphohomocholine increase the radiation response of human glioblastoma cell lines in vitro. *Radiation Oncology (London, England)*, *1*, 6. http://doi.org/10.1186/1748-717X-1-6
184. Ruiz-Ruiz, M. C., & Lopez-Rivas, A. (1999). p53-mediated up-regulation of CD95 is not involved in genotoxic drug-induced apoptosis of human breast tumor cells. *Cell Death and Differentiation*, *6*(3), 271–280. http://doi.org/10.1038/sj.cdd.4400490
185. Russo, P., Malacarne, D., Falugi, C., Trombino, S., & O'Connor, P. M. (2002). RPR-115135, a farnesyltransferase inhibitor, increases 5-FU- cytotoxicity in ten human colon cancer cell lines: role of p53. *International Journal of Cancer*, *100*(3), 266–275. http://doi.org/10.1002/ijc.10461
186. Sain, N., Krishnan, B., Ormerod, M. G., De Rienzo, A., Liu, W. M., Kaye, S. B., … Jackman, A. L. (2006). Potentiation of paclitaxel activity by the HSP90 inhibitor 17-allylamino-17-demethoxygeldanamycin in human ovarian carcinoma cell lines with high levels of activated AKT. *Molecular Cancer Therapeutics*, *5*(5), 1197–1208. http://doi.org/10.1158/1535-7163.MCT-05-0445

187. Sambrook J, Fritsch E F, Maniatis T. Molecular Cloning: A I.aboratory Manual. New York: Cold Spring Harbor l.aboratory Press, 1989. (For Q-factor Q=E(A+B)/(EA+(1--EA)EB))

188. Sangai, T., Akcakanat, A., Chen, H., Tarco, E., Wu, Y., Do, K.-A., … Meric-Bernstam, F. (2012). Biomarkers of response to Akt inhibitor MK-2206 in breast cancer. *Clinical Cancer Research : An Official Journal of the American Association for Cancer Research*, *18*(20), 5816–5828. http://doi.org/10.1158/1078-0432.CCR-12-1141

189. Schaefer, G., Haber, L., Crocker, L. M., Shia, S., Shao, L., Dowbenko, D., … Eigenbrot, C. (2011). A two-in-one antibody against HER3 and EGFR has superior inhibitory activity compared with monospecific antibodies. *Cancer Cell*, *20*(4), 472–486. http://doi.org/10.1016/j.ccr.2011.09.003

190. Seoane, S., Montero, J. C., Ocana, A., & Pandiella, A. (2010). Effect of multikinase inhibitors on caspase-independent cell death and DNA damage in HER2-overexpressing breast cancer cells. *Journal of the National Cancer Institute*, *102*(18), 1432–1446. http://doi.org/10.1093/jnci/djq315

191. Shalinsky, D. R., Slovak, M. L., & Howell, S. B. (1991). Modulation of vinblastine sensitivity by dipyridamole in multidrug resistant fibrosarcoma cells lacking mdr1 expression. *British Journal of Cancer*, *64*(4), 705–709.

192. Shang, D., Liu, Y., Matsui, Y., Ito, N., Nishiyama, H., Kamoto, T., & Ogawa, O. (2008). Demethylating agent 5-aza-2’-deoxycytidine enhances susceptibility of bladder transitional cell carcinoma to Cisplatin. *Urology*, *71*(6), 1220–1225. http://doi.org/10.1016/j.urology.2007.11.029

193. Shen, J., Zheng, H., Ruan, J., Fang, W., Li, A., Tian, G., … Zhao, P. (2013). Autophagy inhibition induces enhanced proapoptotic effects of ZD6474 in glioblastoma. *British Journal of Cancer*, *109*(1), 164–171. http://doi.org/10.1038/bjc.2013.306

194. Shingu, T., Yamada, K., Hara, N., Moritake, K., Osago, H., Terashima, M., … Tsuchiya, M. (2003). Synergistic augmentation of antimicrotubule agent-induced cytotoxicity by a phosphoinositide 3-kinase inhibitor in human malignant glioma cells. *Cancer Research*, *63*(14), 4044–4047.

195. Simpson, W. G., Tseng, M. T., Anderson, K. C., & Harty, J. I. (1984). Verapamil enhancement of chemotherapeutic efficacy in human bladder cancer cells. *The Journal of Urology*, *132*(3), 574–576.

196. Sims, J. T., Ganguly, S., Fiore, L. S., Holler, C. J., Park, E.-S., & Plattner, R. (2009). STI571 sensitizes breast cancer cells to 5-fluorouracil, cisplatin and camptothecin in a cell type-specific manner. *Biochemical Pharmacology*, *78*(3), 249–260. http://doi.org/10.1016/j.bcp.2009.04.007

197. Soldi, R., Cohen, A. L., Cheng, L., Sun, Y., Moos, P. J., & Bild, A. H. (2013). A genomic approach to predict synergistic combinations for breast cancer treatment. *The Pharmacogenomics Journal*, *13*(1), 94–104. http://doi.org/10.1038/tpj.2011.48

198. Sonpavde, G., Jian, W., Liu, H., Wu, M.-F., Shen, S. S., & Lerner, S. P. (2009). Sunitinib malate is active against human urothelial carcinoma and enhances the activity of cisplatin in a preclinical model. *Urologic Oncology*, *27*(4), 391–399. http://doi.org/10.1016/j.urolonc.2008.03.017

199. Srirangam, A., Milani, M., Mitra, R., Guo, Z., Rodriguez, M., Kathuria, H., … Potter, D. A. (2011). The human immunodeficiency virus protease inhibitor ritonavir inhibits lung

cancer cells, in part, by inhibition of survivin. *Journal of Thoracic Oncology : Official Publication of the International Association for the Study of Lung Cancer*, *6*(4), 661–670. http://doi.org/10.1097/JTO.0b013e31820c9e3c

200. Stehlik, P., Paulikova, H., & Hunakova, L. (2010). Synthetic isothiocyanate indole-3-ethyl isothiocyanate (homoITC) enhances sensitivity of human ovarian carcinoma cell lines A2780 and A2780/CP to cisplatin. *Neoplasma*, *57*(5), 473–481.

201. Sugiyama, K., Shimizu, M., Akiyama, T., Ishida, H., Okabe, M., Tamaoki, T., & Akinaga, S. (1998). Combined effect of navelbine with medroxyprogesterone acetate against human breast carcinoma MCF-7 cells in vitro. *British Journal of Cancer*, *77*(11), 1737–1743.

202. Sun, Y., Sheng, Z., Ma, C., Tang, K., Zhu, R., Wu, Z., … Cao, Z. (2015). Combining genomic and network characteristics for extended capability in predicting synergistic drugs for cancer. *Nature Communications*, *6*, 8481. http://doi.org/10.1038/ncomms9481

203. Takeshita, T., Wu, W., Koike, A., Fukuda, M., & Ohta, T. (2009). Perturbation of DNA repair pathways by proteasome inhibitors corresponds to enhanced chemosensitivity of cells to DNA damage-inducing agents. *Cancer Chemotherapy and Pharmacology*, *64*(5), 1039–1046. http://doi.org/10.1007/s00280-009-0961-5

204. Tang, Y., Hamed, H. A., Poklepovic, A., Dai, Y., Grant, S., & Dent, P. (2012). Poly(ADP-ribose) polymerase 1 modulates the lethality of CHK1 inhibitors in mammary tumors. *Molecular Pharmacology*, *82*(2), 322–332. http://doi.org/10.1124/mol.112.078907

205. Tarasenko, N., Cutts, S. M., Phillips, D. R., Berkovitch-Luria, G., Bardugo-Nissim, E., Weitman, M., … Rephaeli, A. (2014). A novel valproic acid prodrug as an anticancer agent that enhances doxorubicin anticancer activity and protects normal cells against its toxicity

in vitro and in vivo. *Biochemical Pharmacology*, *88*(2), 158–168. http://doi.org/10.1016/j.bcp.2014.01.023

206. Taylor-Harding, B., Orsulic, S., Karlan, B. Y., & Li, A. J. (2010). Fluvastatin and cisplatin demonstrate synergistic cytotoxicity in epithelial ovarian cancer cells. *Gynecologic Oncology*, *119*(3), 549–556. http://doi.org/10.1016/j.ygyno.2010.08.017

207. Teicher, B. A., Menon, K., Alvarez, E., Galbreath, E., Shih, C., & Faul, M. M. (2001). Antiangiogenic and antitumor effects of a protein kinase Cbeta inhibitor in human HT-29 colon carcinoma and human CaKi1 renal cell carcinoma xenografts. *Anticancer Research*, *21*(5), 3175–3184.

208. Terashima, M., Sakai, K., Togashi, Y., Hayashi, H., De Velasco, M. A., Tsurutani, J., & Nishio, K. (2014). Synergistic antitumor effects of S-1 with eribulin in vitro and in vivo for triple-negative breast cancer cell lines. *SpringerPlus*, *3*, 417. http://doi.org/10.1186/2193-1801-3-417

209. Thakur, A., Joshi, N., Shanmugam, T., & Banerjee, R. (2013). Proapoptotic miltefosine nanovesicles show synergism with paclitaxel: Implications for glioblastoma multiforme therapy. *Cancer Letters*, *334*(2), 274–283. http://doi.org/10.1016/j.canlet.2012.08.022

210. Tiwary, R., Yu, W., Sanders, B. G., & Kline, K. (2011). alpha-TEA cooperates with chemotherapeutic agents to induce apoptosis of p53 mutant, triple-negative human breast cancer cells via activating p73. *Breast Cancer Research : BCR*, *13*(1), R1. http://doi.org/10.1186/bcr2801

211. Trendowski, M., Christen, T. D., Andonova, A. A., Narampanawe, B., Thibaud, A., Kusang, T., & Fondy, T. P. (2015). Effects of mTOR inhibitors and cytoskeletal-directed agents alone

and in combination against normal and neoplastic hematopoietic cells in vitro. *Investigational New Drugs*, *33*(6), 1162–1174. http://doi.org/10.1007/s10637-015-0294-7

212. Tsakalozou, E., Eckman, A. M., & Bae, Y. (2012). Combination effects of docetaxel and Doxorubicin in hormone-refractory prostate cancer cells. *Biochemistry Research International*, *2012*, 832059. http://doi.org/10.1155/2012/832059

213. Tseng, P.-H., Wang, Y.-C., Weng, S.-C., Weng, J.-R., Chen, C.-S., Brueggemeier, R. W., … Chen, C.-S. (2006). Overcoming trastuzumab resistance in HER2-overexpressing breast cancer cells by using a novel celecoxib-derived phosphoinositide-dependent kinase-1 inhibitor. *Molecular Pharmacology*, *70*(5), 1534–1541. http://doi.org/10.1124/mol.106.023911

214. Vega, M. I., Huerta-Yepez, S., Jazirehi, A. R., Garban, H., & Bonavida, B. (2005). Rituximab (chimeric anti-CD20) sensitizes B-NHL cell lines to Fas-induced apoptosis. *Oncogene*, *24*(55), 8114–8127. http://doi.org/10.1038/sj.onc.1208954

215. Viale, M., Pastrone, I., Pellecchia, C., Vannozzi, M. O., Cafaggi, S., & Esposito, M. (1998). Combination of cisplatin-procaine complex DPR with anticancer drugs increases cytotoxicity against ovarian cancer cell lines. *Anti-Cancer Drugs*, *9*(5), 457–463.

216. Vinod, B. S., Antony, J., Nair, H. H., Puliyappadamba, V. T., Saikia, M., Narayanan, S. S., … Anto, R. J. (2013). Mechanistic evaluation of the signaling events regulating curcumin-mediated chemosensitization of breast cancer cells to 5-fluorouracil. *Cell Death & Disease*, *4*, e505. http://doi.org/10.1038/cddis.2013.26

217. Wainberg, Z. A., Anghel, A., Rogers, A. M., Desai, A. J., Kalous, O., Conklin, D., … Finn, R. S. (2013). Inhibition of HSP90 with AUY922 induces synergy in HER2-amplified trastuzumab-

resistant breast and gastric cancer. *Molecular Cancer Therapeutics*, *12*(4), 509–519. http://doi.org/10.1158/1535-7163.MCT-12-0507

218. Wang, J., Chen, J., Miller, D. D., & Li, W. (2014). Synergistic combination of novel tubulin inhibitor ABI-274 and vemurafenib overcome vemurafenib acquired resistance in BRAFV600E melanoma. *Molecular Cancer Therapeutics*, *13*(1), 16–26. http://doi.org/10.1158/1535-7163.MCT-13-0212

219. Wang, Y. A., Johnson, S. K., Brown, B. L., McCarragher, L. M., Al-Sakkaf, K., Royds, J. A., & Dobson, P. R. M. (2008). Enhanced anti-cancer effect of a phosphatidylinositol-3 kinase inhibitor and doxorubicin on human breast epithelial cell lines with different p53 and oestrogen receptor status. *International Journal of Cancer*, *123*(7), 1536–1544. http://doi.org/10.1002/ijc.23671

220. Wang, Y., Wang, H., Zhang, W., Shao, C., Xu, P., Shi, C. H., … Zhang, Y. T. (2013). Genistein sensitizes bladder cancer cells to HCPT treatment in vitro and in vivo via ATM/NF-kappaB/IKK pathway-induced apoptosis. *PloS One*, *8*(1), e50175. http://doi.org/10.1371/journal.pone.0050175

221. Wang, Y., Yang, Z., & Zhao, X. (2010). Honokiol induces paraptosis and apoptosis and exhibits schedule-dependent synergy in combination with imatinib in human leukemia cells. *Toxicology Mechanisms and Methods*, *20*(5), 234–241. http://doi.org/10.3109/15376511003758831

222. Wawruszak, A., Luszczki, J. J., Grabarska, A., Gumbarewicz, E., Dmoszynska-Graniczka, M., Polberg, K., & Stepulak, A. (2015). Assessment of Interactions between Cisplatin and Two Histone Deacetylase Inhibitors in MCF7, T47D and MDA-MB-231 Human Breast Cancer Cell

Lines - An Isobolographic Analysis. *PloS One*, *10*(11), e0143013. http://doi.org/10.1371/journal.pone.0143013

223. Weisberg, E., Catley, L., Kujawa, J., Atadja, P., Remiszewski, S., Fuerst, P., … Griffin, J. D. (2004). Histone deacetylase inhibitor NVP-LAQ824 has significant activity against myeloid leukemia cells in vitro and in vivo. *Leukemia*, *18*(12), 1951–1963. http://doi.org/10.1038/sj.leu.2403519

224. Wesierska-Gadek, J., Zulehner, N., Ferk, F., Skladanowski, A., Komina, O., & Maurer, M. (2012). PARP inhibition potentiates the cytotoxic activity of C-1305, a selective inhibitor of topoisomerase II, in human BRCA1-positive breast cancer cells. *Biochemical Pharmacology*, *84*(10), 1318–1331. http://doi.org/10.1016/j.bcp.2012.07.024

225. West, N. W., Garcia-Vargas, A., Chalfant, C. E., & Park, M. A. (2013). OSU-03012 sensitizes breast cancers to lapatinib-induced cell killing: a role for Nck1 but not Nck2. *BMC Cancer*, *13*, 256. http://doi.org/10.1186/1471-2407-13-256

226. Winterhoff, B., Freyer, L., Hammond, E., Giri, S., Mondal, S., Roy, D., … Shridhar, V. (2015). PG545 enhances anti-cancer activity of chemotherapy in ovarian models and increases surrogate biomarkers such as VEGF in preclinical and clinical plasma samples. *European Journal of Cancer (Oxford, England : 1990)*, *51*(7), 879–892. http://doi.org/10.1016/j.ejca.2015.02.007

227. Witters, L., Scherle, P., Friedman, S., Fridman, J., Caulder, E., Newton, R., & Lipton, A. (2008). Synergistic inhibition with a dual epidermal growth factor receptor/HER-2/neu tyrosine kinase inhibitor and a disintegrin and metalloprotease inhibitor. *Cancer Research*, *68*(17), 7083–7089. http://doi.org/10.1158/0008-5472.CAN-08-0739

228. Wong, F. Y., Liem, N., Xie, C., Yan, F. L., Wong, W. C., Wang, L., & Yong, W.-P. (2012). Combination therapy with gossypol reveals synergism against gemcitabine resistance in cancer cells with high BCL-2 expression. *PloS One*, *7*(12), e50786. http://doi.org/10.1371/journal.pone.0050786

229. Wu, J., Wang, W., Shao, Q., Xiao, G., Cheng, J., Yuan, Y., & Zhang, M. (2014). Irradiation facilitates the inhibitory effect of the heat shock protein 90 inhibitor NVP-BEP800 on the proliferation of malignant glioblastoma cells through attenuation of the upregulation of heat shock protein 70. *Experimental and Therapeutic Medicine*, *8*(3), 893–898. http://doi.org/10.3892/etm.2014.1800

230. Xing, L., Zhang, Z., Xu, Y., Zhang, H., & Liu, J. (2004). The effects of nimesulide combined with cisplatin on lung cancer. *Journal of Huazhong University of Science and Technology. Medical Sciences = Hua Zhong Ke Ji Da Xue Xue Bao. Yi Xue Ying De Wen Ban = Huazhong Keji Daxue Xuebao. Yixue Yingdewen Ban*, *24*(2), 120–123.

231. Yan, H., Wang, Y.-C., Li, D., Wang, Y., Liu, W., Wu, Y.-L., & Chen, G.-Q. (2007). Arsenic trioxide and proteasome inhibitor bortezomib synergistically induce apoptosis in leukemic cells: the role of protein kinase Cdelta. *Leukemia*, *21*(7), 1488–1495. http://doi.org/10.1038/sj.leu.2404735

232. Yan, K.-H., Yao, C.-J., Chang, H.-Y., Lai, G.-M., Cheng, A.-L., & Chuang, S.-E. (2010). The synergistic anticancer effect of troglitazone combined with aspirin causes cell cycle arrest and apoptosis in human lung cancer cells. *Molecular Carcinogenesis*, *49*(3), 235–246. http://doi.org/10.1002/mc.20593

233. Yang, E., Boire, A., Agarwal, A., Nguyen, N., O'Callaghan, K., Tu, P., … Covic, L. (2009). Blockade of PAR1 signaling with cell-penetrating pepducins inhibits Akt survival pathways

in breast cancer cells and suppresses tumor survival and metastasis. *Cancer Research*, *69*(15), 6223–6231. http://doi.org/10.1158/0008-5472.CAN-09-0187

234. Yang, Z., Lee, M.-J., Zhao, Y., & Yang, C. S. (2012). Metabolism of tocotrienols in animals and synergistic inhibitory actions of tocotrienols with atorvastatin in cancer cells. *Genes & Nutrition*, *7*(1), 11–18. http://doi.org/10.1007/s12263-011-0233-y
235. Yip, K. W., Zhang, Z., Sakemura-Nakatsugawa, N., Huang, J.-W., Vu, N. M., Chiang, Y.-K., … Liu, F.-F. (2014). A porphodimethene chemical inhibitor of uroporphyrinogen decarboxylase. *PloS One*, *9*(2), e89889. http://doi.org/10.1371/journal.pone.0089889
236. Yu, C., Liu, S.-L., Qi, M.-H., Zou, X., Wu, J., & Zhang, J. (2015). Herbal medicine Guan Chang Fu Fang enhances 5-fluorouracil cytotoxicity and affects drug-associated genes in human colorectal carcinoma cells. *Oncology Letters*, *9*(2), 701–708. http://doi.org/10.3892/ol.2014.2766
237. Zhang, J., Li, B., Wu, H., Ou, J., Wei, R., Liu, J., … Liang, A. (2016). Synergistic action of 5Z-7-oxozeaenol and bortezomib in inducing apoptosis of Burkitt lymphoma cell line Daudi. *Tumour Biology : The Journal of the International Society for Oncodevelopmental Biology and Medicine*, *37*(1), 531–539. http://doi.org/10.1007/s13277-015-3832-1
238. Zhang, L., Chen, Z., Yang, K., Liu, C., Gao, J., & Qian, F. (2015). beta-Lapachone and Paclitaxel Combination Micelles with Improved Drug Encapsulation and Therapeutic Synergy as Novel Nanotherapeutics for NQO1-Targeted Cancer Therapy. *Molecular Pharmaceutics*, *12*(11), 3999–4010. http://doi.org/10.1021/acs.molpharmaceut.5b00448
239. Zhang, Q.-Y., Mao, J.-H., Liu, P., Huang, Q.-H., Lu, J., Xie, Y.-Y., … Chen, Z. (2009). A systems biology understanding of the synergistic effects of arsenic sulfide and Imatinib in BCR/ABL-

associated leukemia. *Proceedings of the National Academy of Sciences of the United States of America*, *106*(9), 3378–3383. http://doi.org/10.1073/pnas.0813142106

240. Zhang, Y., Wu, Y., Wu, D., Tashiro, S., Onodera, S., & Ikejima, T. (2009). NF-kappab facilitates oridonin-induced apoptosis and autophagy in HT1080 cells through a p53-mediated pathway. *Archives of Biochemistry and Biophysics*, *489*(1–2), 25–33. http://doi.org/10.1016/j.abb.2009.07.017

241. Zhao, G.-X., Xu, L.-H., Pan, H., Lin, Q.-R., Huang, M.-Y., Cai, J.-Y., … He, X.-H. (2015). The BH3-mimetic gossypol and noncytotoxic doses of valproic acid induce apoptosis by suppressing cyclin-A2/Akt/FOXO3a signaling. *Oncotarget*, *6*(36), 38952–38966. http://doi.org/10.18632/oncotarget.5731

242. Zhao, Y., Liu, H., Liu, Z., Ding, Y., Ledoux, S. P., Wilson, G. L., … Tan, M. (2011). Overcoming trastuzumab resistance in breast cancer by targeting dysregulated glucose metabolism. *Cancer Research*, *71*(13), 4585–4597. http://doi.org/10.1158/0008-5472.CAN-11-0127

243. Zheng, F.-M., Long, Z.-J., Hou, Z.-J., Luo, Y., Xu, L.-Z., Xia, J.-L., … Liu, Q. (2014). A novel small molecule aurora kinase inhibitor attenuates breast tumor-initiating cells and overcomes drug resistance. *Molecular Cancer Therapeutics*, *13*(8), 1991–2003. http://doi.org/10.1158/1535-7163.MCT-13-1029

244. Zhou, N., Singh, K., Mir, M. C., Parker, Y., Lindner, D., Dreicer, R., … Hansel, D. E. (2013). The investigational Aurora kinase A inhibitor MLN8237 induces defects in cell viability and cell-cycle progression in malignant bladder cancer cells in vitro and in vivo. *Clinical Cancer Research : An Official Journal of the American Association for Cancer Research*, *19*(7), 1717–28. http://doi.org/10.1158/1078-0432.CCR-12-2383

245. Zou, B., Li, Q. Q., Zhao, J., Li, J. M., Cuff, C. F., & Reed, E. (2013). beta-Elemene and taxanes synergistically induce cytotoxicity and inhibit proliferation in ovarian cancer and other tumor cells. *Anticancer Research*, *33*(3), 929–940.
