# Supplementary material for: A cancer drug atlas enables synergistic targeting of independent drug vulnerabilities
Source: Nat Commun. 2020 Jun 10;11:2935. doi: 10.1038/s41467-020-16735-2 (PMC7287046; doi:10.1038/s41467-020-16735-2)
Supplement: Supplementary file 3 — Description of Additional Supplementary Files [file 41467_2020_16735_MOESM3_ESM.pdf]

Description of Additional Supplementary Files

File Name: Supplementary Data 1
Description: **Overview sources.** Overview of data sources used for the analyses

File Name: Supplementary Data 2
Description: **Curated data.** List of 483 manually curated drug-drug pairs from the literature

File Name: Supplementary Data 3A
Description: **IC50s glioma cell lines.** IC50 and AUCs of glioma cell lines and drugs used for this study

File Name: Supplementary Data 3B
Description: **Dual synergy combination index**. Combination indexes (CIs) as determined when using 30 drug pairs onto 9 glioma cell lines

File Name: Supplementary Data 3C
Description: **Synergy metrics.** Comparing the combination index to Loewe, Bliss and HSA metrics

File Name: Supplementary Data 3D
Description: **Synergy predictions.** List of drug atlas top predicted combinations against 423 cell lines

File Name: Supplementary Data 4
Description: **Triple synergy combination indexes.** Triple combination indexes (CIs), Osimeretinib, AZD2014, Docetaxel pairs onto 9 glioma cell lines

File Name: Supplementary Data 5
Description: **In vivo efficacy.** Comparison of the median survival rates of the in vivo experiments
